# Time to put the mammillothalamic pathway into context

**DOI:** 10.1016/j.neubiorev.2020.11.031

**Published:** 2021-02

**Authors:** Christopher M. Dillingham, Michal M. Milczarek, James C. Perry, Seralynne D. Vann

**Affiliations:** School of Psychology, Neuroscience and Mental Health Research Institute, Cardiff University, Cardiff, CF10 3AT, UK

**Keywords:** Amnesia, Anterior thalamic nuclei, Diencephalon, Hippocampus, Mammillary bodies, Memory, Retrosplenial cortex

## Abstract

The medial diencephalon, in particular the mammillary bodies and anterior thalamic nuclei, has long been linked to memory and amnesia. The mammillary bodies provide a dense input into the anterior thalamic nuclei, via the mammillothalamic tract. In both animal models, and in patients, lesions of the mammillary bodies, mammillothalamic tract and anterior thalamic nuclei all produce severe impairments in temporal and contextual memory, yet it is uncertain why these regions are critical. Mounting evidence from electrophysiological and neural imaging studies suggests that mammillothalamic projections exercise considerable distal influence over thalamo-cortical and hippocampo-cortical interactions. Here, we outline how damage to the mammillary body-anterior thalamic axis, in both patients and animal models, disrupts behavioural performance on tasks that relate to contextual (“where”) and temporal (“when”) processing. Focusing on the medial mammillary nuclei as a possible ‘theta-generator’ (through their interconnections with the ventral tegmental nucleus of Gudden) we discuss how the mammillary body-anterior thalamic pathway may contribute to the mechanisms via which the hippocampus and neocortex encode representations of experience.

## Highlights

• The mammillothalamic pathway plays a key role in temporal and contextual memory.
• The mammillothalamic pathway mediates hippocampo-cortical functions.
• The mammillothalamic pathway plays an important role in oscillatory co- ordination.

## 1 Introduction

Memory is thought to arise from the integration of multiple processing streams subserved by several anatomical brain circuits. The anterior thalamic nuclei (ATN), which are positioned at the interface between hippocampal, cortical, and subcortical mnemonic networks, are posited to play a key role in cognition and memory (Dillingham et al., 2015a). Yet, despite significant advances in elucidating the roles of the hippocampus and cortex, still relatively little is known about the specific contributions of subcortical inputs, including the ATN and mammillary bodies (MBs). The MBs, a complex of small hypothalamic nuclei, constitute a major excitatory drive for the ATN, issuing dense unidirectional projections via the mammillothalamic tract (MTT). Damage to either the MBs, the MTT, or the ATN produce recollective memory impairments in humans (Carlesimo et al., 2007; Cipolotti et al., 2008; Gentilini et al., 1987; Harding et al., 2000; Hildebrandt et al., 2001; Kapur et al., 1996; Tanaka et al., 1997; Van der Werf et al., 2003) and spatial memory impairments in rodents (e.g. Byatt and Dalrymple-Alford, 1996; Field et al., 1978; Rosenstock et al., 1977; Vann and Aggleton, 2003). Furthermore, the degree of functional coupling between the MBs and the ATN has been shown to predict the severity of cognitive decline in Wernicke’s encephalopathy (Kim et al., 2009). Together, these findings suggest that the MB-ATN axis forms a vital component of the wider mnemonic circuitry.

Much of our knowledge about medial diencephalic function has derived from studies involving patients with Korsakoff syndrome, a core feature of which is damage to the MBs and the ATN. In addition to a general memory impairment, one of the first specific deficits to be noted in this patient group was abnormal temporal memory (Kessels and Kopelman, 2012; Korsakoff et al., 1996). The temporal features of an event are a main component of episodic memory, i.e., the memory of what, where and *when*. The original observation of impoverished temporal memory has been extended over the years to include context more generally, i.e., the background environment, typically encompassing geometric and/or visuospatial features. As such, the medial diencephalon may be important for encoding contextual cues (Mayes et al., 1985), or for binding features/items with these contextual cues (Chalfonte et al., 1996). An impaired representation of context is likely to further disrupt the effective encoding of specific events. This is because, under normal circumstances, contextual cues allocated to specific memories help reduce overlapping mnemonic representations and thus reduce the likelihood of similar events interfering with each other. In addition to the loss of contextual detail in newly acquired memories, there is also evidence that memories acquired prior to the onset of medial diencephalic pathology lack spatio-temporal detail (Hodges and McCarthy, 1993). This loss of both temporal and contextual information means individuals can lose access to their own personal timelines as memories are no longer grounded by circumstantial detail.

**Fig. 1:**
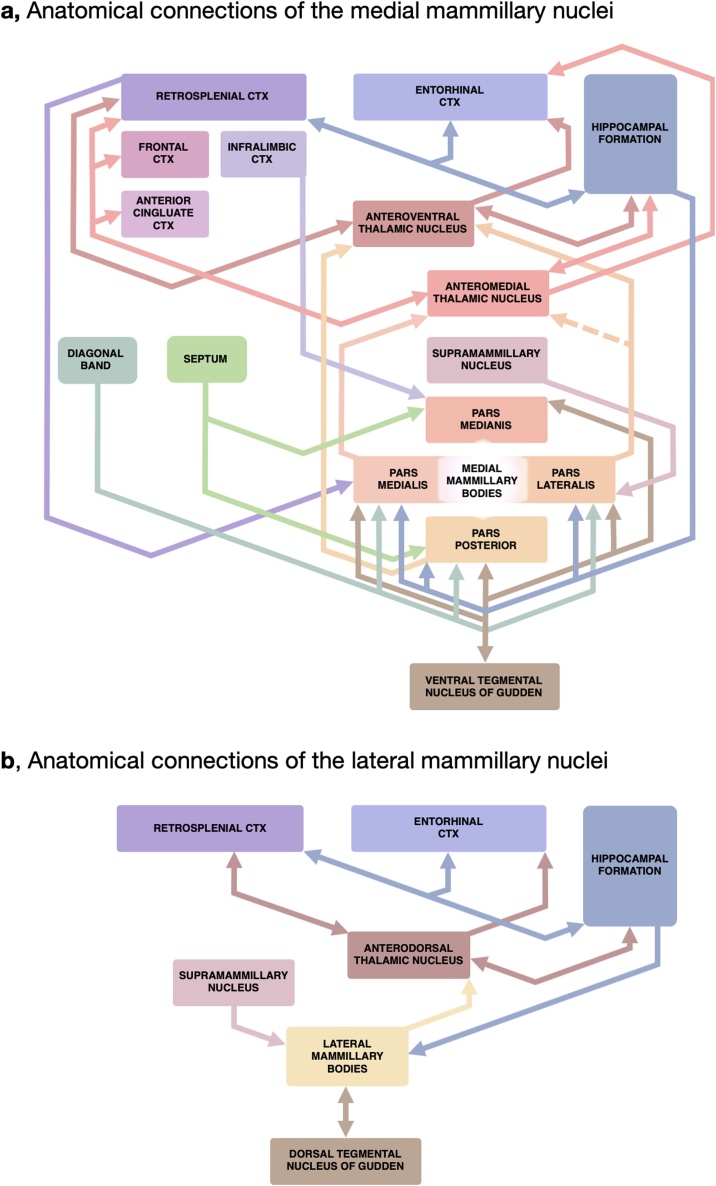
Main anatomical connections associated with the mammillary body-anterior thalamic pathway. The mammillary bodies receive topographically organised inputs from midbrain, subcortical, and cortical brain regions and project to the anterior thalamic nuclei. a, Anatomical connections of the medial mammillary nuclei. The principle output from the medial nuclei constitute topographical projections from pars medialis and pars lateralis sub-nuclei to the anteromedial and anteroventral thalamic nuclei, respectively. Hippocampal efferents derive predominantly from the dorsal, intermediate and ventral subiculum. The ventral tegmental nuclei of Gudden send a dense GABAergic projection to the medial mammillary bodies, reciprocated by medial mammillary projections. This recurrent circuit is thought to aid the generation of theta rhythmicity (see Section 3.1). b, Anatomical connections of the lateral mammillary nuclei. Reciprocal connections between the lateral mammillary bodies and the dorsal tegmental nuclei of Gudden constitute the generative pathway of the vestibular head direction system. Thalamic projections of the lateral mammillary nuclei are exclusively to the anterodorsal thalamic nuclei while hippocampal inputs to the lateral mammillary nuclei arise dominantly from the postsubiculum of the hippocampal formation. Dashed line in A represents a possible but unconfirmed projection (see Section 2.2.2). The connections depicted are for the rat; while the general pattern of connections is similar in the primate, there are some differences in specific subregion connectivity. CTX – cortex.

While many of the mnemonic impairments associated with diencephalic amnesia have been well-described, the neural processes underlying normal medial diencephalic function have been more difficult to gauge. For most of the 20^th^ century, the MBs and the ATN were considered to be merely a part of an 'extended hippocampal system' whose main function was to relay hippocampal signals to the cortex. While this model accurately reflects connectivity of the major anatomical pathways (including subicular inputs to the MBs via the fornix, MB projections to the ATN via the MTT and, finally, ATN efferents to both the hippocampus and the neocortex; see Fig. 1), it does not account for processing of information by the MBs themselves, including their specific inputs from the tegmentum. Indeed, mounting evidence suggests that regarding the medial diencephalon (including the MBs and ATN) as a hippocampal relay is an oversimplification (see Sherman and Guillery, 1998; Wolff and Vann, 2019). On the contrary, it is becoming apparent that under certain conditions MBs can exert long-range modulatory control over hippocampo-cortical activity by integrating and propagating signals from their subcortical inputs (Dillingham et al., 2019). Indeed, the MBs receive a number of extra-hippocampal afferents (including the tegmental nuclei of Gudden, medial septum and supramammillary nucleus) which again suggests they process more diverse information streams. This idea is further supported by behavioural evidence as disconnection of hippocampal inputs to the MBs fails to produce deficits as severe as those following MB or MTT lesions (Roy et al., 2017; Tonkiss and Rawlins, 1992; Vann, 2013; Vann et al., 2011) reinforcing the importance of these non-hippocampal inputs.

This review will principally focus on the contributions of the MB-ATN axis to temporal and contextual memory and discuss some more recent evidence elucidating the possible underlying neural mechanisms. To do so, we will consider the links between the anatomy, connectivity, and physiology of the MBs, especially in the context of loss of function upon deliberate (animal lesion models), or incidental damage (clinical data). At least two distinct MB processing streams exist: the relatively well-characterised lateral head direction pathway [Fig. 1b; for recent reviews see Dudchenko et al., 2019 and Dillingham and Vann (2019)] and the medial, theta-related pathway (Fig. 1a). This review will predominantly expound on the latter as the medial system enables closer anatomical comparisons across species and provides a more relevant context for clinical findings (Casatti et al., 2002). However, one caveat relating to the interpretation of MB lesion effects in animals, and, to an extent, clinical findings, is that pathology typically includes both lateral and medial nuclei (and can include some supramammillary damage). ATN lesions also typically include all three nuclei. In contrast, rat studies involving MTT lesions appear to predominantly disrupt medial MB-ATN fibres (Vann and Albasser, 2009). Consequently, findings from some studies may reflect the involvement of both the medial and the lateral MB systems, while others will more specific to the medial MB system. The focus of this review will be on the potential mechanisms by which the medial mammillothalamic pathway may contribute to the thalamic, hippocampal and cortical processes, which are thought to support the representation of contextual and temporal memory. However, the possible contribution of the lateral stream will also be considered in Section 6.

## 2 Nomenclature, cytoarchitecture, and anatomical connectivity

### 2.1 Nomenclature and cytoarchitecture

The MBs are a complex of nuclei occupying the ventral floor of the posterior hypothalamus, appearing as a pair of spherical protrusions from the underside of the brain. Most anatomical and physiological characteristics of the MBs have emerged from detailed studies of the rodent diencephalon which shares a good degree of homology with its human counterpart. In rodents, the MBs comprise the smaller lateral nuclei, which contain relatively large and densely packed fusiform/reticular neurons, and the more prominent medial nuclei, containing more loosely packed fusiform cells (Babmindra et al., 1979).

The rodent medial MBs can be further split into several subdivisions: pars lateralis, pars medialis, pars basalis, pars medianus and pars posterior (reflecting differences in their cytoarchitecture and thalamic connectivity). The rostral boundary of the MBs is defined by the poorly differentiated pre-mammillary nucleus, while dorsally, the MBs are limited by horizontally oriented axonal fibres coursing beneath the supramammillary nucleus. The MBs’ morphogenesis relies on a tightly orchestrated cascade of genetic events (Alvarez-Bolado et al., 2000; Marion et al., 2005; Szabo et al., 2015; Tsuchiya et al., 2009; Valverde et al., 2000; Zappala et al., 2007), disruption of which can result in deficits akin to those following targeted ablation of the MBs or the MTT (Radyushkin et al., 2005).

### 2.2 Anatomical connectivity

#### 2.2.1 Mammillary body afferent connections

Both lateral and medial MBs receive their principal inputs from two regions: the hippocampal formation and Gudden’s tegmental nuclei (Fig. 1). The hippocampal projections travel to the MBs via the fornix. All medial MB subregions other than pars medianus are innervated by the subiculum with inputs originating in the dorsal, intermediate and ventral subiculum (Allen and Hopkins, 1989; Huang et al., 2017). Interestingly, a large proportion of dorsal subicular neurons that project to the medial MBs send collaterals to the entorhinal cortex (as much as ∼80 %; Donovan and Wyss, 1983; Roy et al., 2017), while approximately 50 % of subicular neurons projecting to the retrosplenial cortex send collateral projections to the MBs (Cembrowski et al., 2018; Kinnavane et al., 2018). Such an arrangement could provide a mechanism to co-ordinate activity across distal regions but could also result in some redundancy within the system and perhaps explain the relatively mild effects of hippocampal-MB disconnection (Roy et al., 2017; Tonkiss and Rawlins, 1992; Vann, 2013; Vann et al., 2011). In addition to inputs from the subicular complex, pars lateralis of the medial MBs is also innervated by the medial entorhinal cortex (Shibata, 1988).

In terms of subcortical connections, the ventral tegmental nucleus of Gudden (VTg) innervates all of the medial MB subdivisions in a topographically ordered manner (Allen and Hopkins, 1989). Whereas subicular projections provide a major excitatory drive (Roy et al., 2017; Wright et al., 2010), VTg afferents exert a tonic inhibitory influence over the medial MBs (Kocsis et al., 2001), forming about 80 % of all inhibitory (symmetric) synapses (Hayakawa and Zyo, 1991). Consistently, a high proportion of medial MB-projecting VTg neurons express parvalbumin (Dillingham et al., 2015b) and GAD (Wirtshafter and Stratford, 1993) such that pharmacological inactivation of VTg leads to enhanced activity of the medial MBs (as well as upstream targets; Shim and Wirtshafter, 1996). The presence of GABA, as well as neurotensin and leu-enkephalin have been detected in VTg cells projecting to the MBs (Gonzalo-Ruiz et al., 1999). In addition to these dense connections, more diffuse inputs to the medial MBs arise from the dorsal peduncular cortex, infralimbic cortex (Allen and Hopkins, 1989; Mathiasen et al., 2019), and retrosplenial cortex (Shibata, 1989).

#### 2.2.2 Mammillary body efferent connections

The principal efferent projections of the MBs are to the ATN and Gudden’s tegmental nuclei (Fig. 1), the majority of which collateralise to both regions (Hayakawa and Zyo, 1989). Predominantly glutamatergic projections to the ATN arise from all MB subnuclei (Bernstein et al., 2007; Gonzalo-Ruiz et al., 1996): pars medialis projects to the anteromedial thalamic nucleus (AM); pars lateralis predominantly projects to the anteroventral thalamic nucleus (AV) although some studies (Shibata, 1992; Yamadori, 1973) but not others (Seki and Zyo, 1984) also report a projection to AM; both pars basalis and pars posterior subdivisions project exclusively to the anteroventral thalamic nucleus (Shibata, 1992). All medial MB subregions apart from pars lateralis send excitatory projections, via the mammillotegmental tract, to the VTg (van der Kooy et al., 1978). In addition to their projections to the ATN and the VTg, the medial MBs send diffuse projections to the basal pontine nucleus, pontine tegmental nucleus, central grey area of the brainstem, medial septum and the diagonal band of Broca (Shen, 1983).

#### 2.2.3 Mammillary body-anterior thalamic axis and beyond

While the MBs are not in a position to directly affect wider memory networks, they can indirectly influence a number of hippocampo-cortical regions via their projections to the ATN. For example, the MBs have been shown to influence hippocampal, retrosplenial and frontal activity, as demonstrated by extensive changes in immediate early gene expression following MTT lesions (Frizzati et al., 2016; Vann, 2013; Vann and Albasser, 2009), consistent with the principal output targets of the ATN. Therefore, in order to fully appreciate the wider scope of medial MB influence over the brain, it is most informative to further consider anteromedial (AM) and anteroventral (AV) thalamic efferent projections.

The AM projects to frontal and anterior cingulate cortices (de Lima et al., 2017; Hoover and Vertes, 2007; Shibata and Kato, 1993; van Groen et al., 1999). It also issues a dense projection to the dysgranular retrosplenial cortex (Shibata and Kato, 1993; van Groen et al., 1999), a subregion specifically associated with visuo-spatial processing (Hindley et al., 2014a; Powell et al., 2020; Vann and Aggleton, 2005). In addition, AM is in a position to both directly and indirectly affect hippocampal functions via its projections to medial and lateral entorhinal cortex, presubiculum, subiculum (Shibata, 1993a; Shibata and Kato, 1993; van Groen et al., 1999), as well as direct projections to CA1 (de Lima et al., 2017). As such, AM is well-placed to contribute to hippocampal functioning via multiple, converging routes.

With the exception of frontal cortical connections, AV broadly projects to similar areas as AM. AV projects more widely to the retrosplenial cortex innervating both granular and dysgranular subdivisions (Shibata, 1993b; Van Groen and Wyss, 2003). The projections from AV to the hippocampal complex primarily target pre-, para- and postsubiculum while the subiculum and medial entorhinal cortex receive weaker inputs (Shibata, 1993a; van Groen and Wyss, 1990, 1995). Like the AM nucleus, AV is also able to affect the hippocampus proper via multiple routes, i.e., via light projections to the subiculum and then to CA1 (Sun et al., 2019; Xu et al., 2016); but more likely, via the more dense projections to pre- and parasubiculum and from there to medial entorhinal cortex.

## 3 Electrophysiological properties

### 3.1 Medial mammillary nucleus

While the medial MBs comprise a morphologically homogenous population of projection neurons, there is substantial heterogeneity in terms of the electrophysiological properties (Alonso and Llinas, 1992; Kocsis and Vertes, 1997). In both anaesthetised (Kocsis and Vertes, 1994) and awake-behaving animals (Sharp and Turner-Williams, 2005), a large proportion of medial MB units were found to be entrained to theta band (4−12 Hz) oscillations and modulated by running speed. In addition, angular velocity neurons have been recorded from the medial MBs. Experiments using slice preparations have demonstrated that medial MB neurons exhibit calcium-dependent complex bursting (Alonso and Llinas, 1992; Kocsis and Vertes, 1997), suggesting that these responses may be modulating network excitability (Zeldenrust et al., 2018). Like the medial MBs, a large proportion of VTg neurons exhibit complex bursting activity (Bassant and Poindessous-Jazat, 2001), which is particularly evident during REM (paradoxical) sleep. Consistent with the findings from rodents, theta entrained neurons have also been recorded in human MBs (van Rijckevorsel et al., 2005).

Theta band oscillations encode information critical to mnemonic processing across a wide range of diencephalic and cortical brain areas. In addition to being generated intrinsically (through interneuron-pyramidal cell interactions; Amilhon et al., 2015), hippocampal theta can also be driven by external regions including the medial septum (Brandon et al., 2014; Lee et al., 1994) and the supramammillary nucleus. Inactivating these regions results in the disruption of theta in downstream targets, e.g. inactivation of the medial septum abolishes hippocampal theta while lesions of the medial supramammillary nucleus attenuate the frequency of hippocampal theta oscillations (Pan and McNaughton, 1997; Sharp and Koester, 2008).

The medial MBs, through interactions with the VTg, are thought to constitute an independently-driven source of theta capable of modulating activity in downstream regions. Conjoint recordings in the VTg and the hippocampus revealed that the temporal onset of theta bursting activity in VTg preceded that of the hippocampus, often by a matter of seconds in both awake behaving rats (Bassant and Poindessous-Jazat, 2001) and in anaesthetised rats (Kocsis et al., 2001). Following this delayed onset, however, hippocampal and VTg theta oscillations were found to be highly coherent. The mechanisms by which neurons in the VTg, or VTg-MB interactions generate theta are not well understood, however, parallels between the anatomical and neurochemical characteristics of the VTg-MB axis and the medial septum exist. The activity of hippocampal parvalbuminergic interneurons and GABAergic septo-hippocampal inputs are critical to the generation of hippocampal theta (Amilhon et al., 2015). Comparably, parvalbuminergic interneurons in the VTg also comprise a sizeable input to the medial MB (Dillingham et al., 2015b), which may subserve a similar role, potentially through the modulation of intrinsic theta bursting in MB projection neurons. Such an organisation, i.e. a combination of intrinsic excitation (unit bursting) and extrinsic inhibition (parvalbumineric/GABAergic input) is thought to be one mechanism through which network theta activity in other theta-generating circuitry is generated (Colgin, 2013; Hu et al., 2002; Hutcheon and Yarom, 2000; Stark et al., 2013). Accordingly, the medial MBs express genes promoting rhythmic firing behaviour such as T-type calcium channel receptor subunits (Talley et al., 1999) as well as various calcium binding proteins, including parvalbumin, calbindin, calretinin and hippocalcin (Bernstein et al., 2007; Celio, 1990; Fortin and Parent, 1997; Paterlini et al., 2000; Zakowski et al., 2014). In addition, the medial MBs are also subject to diverse neuromodulatory influences, which are likely to support oscillatory activity and, more broadly, their role in memory.

While more recent proposals have suggested medial MB-VTg interactions comprise an independent theta source, the original view was that MB theta was driven via its hippocampal inputs. This line of thinking was supported by a group of studies that assessed the effects of hippocampal theta disruption on MB theta in anaesthetised animals. Abolition of hippocampal theta through inactivation of the medial septum was found to inhibit theta modulation in MB units (Kirk et al., 1996). Theta modulation of medial MB neurons was, however, re-elicited by stimulation of nucleus pontis oralis (RPO), which reinstated hippocampal theta through the RPO-supramammillary-septal pathway (Kirk et al., 1996). While these findings suggest that MB theta entrainment is dependent on septo-hippocampal inputs, it does not account for the finding that sensory-elicited theta, e.g. toe-pinch, reinstated theta modulation in the absence of descending hippocampal inputs. More recently, in awake and behaving rats, abolition of hippocampal theta through inactivation of the medial septum was found to reduce, but not abolish theta oscillatory power in the MBs and, interestingly, increased the dominant frequency of theta in the MBs (Ruan et al., 2017). Given that local field potential recordings are thought to represent the post-synaptic input into the recording site, the residual theta activity observed in the medial MBs may reflect theta generated through interconnections with the VTg.

### 3.2 Relationship with anterior thalamic nuclei electrophysiology

Out of the three anterior thalamic nuclei, the electrophysiological properties of the anterodorsal thalamic nucleus (AD) have been the most extensively examined, with a high proportion of head direction cells reported (Blair et al., 1998). The lateral MB-AD pathway **(**Fig. 1b**)** forms an established element of the head direction network and, significantly, directional encoding in AD is dependent on mammillothalamic, rather than postsubicular inputs (Dillingham and Vann, 2019). In turn, directional activity in hippocampo-cortical regions is dependent on ascending AD projections (but see Clark et al., 2010).

By contrast, the electrophysiological properties of AV and AM nuclei have been less well characterised. The studies available, however, show that a notable proportion of AV and AM neuronal activity is entrained to theta band oscillatory input (Albo et al., 2006; Tsanov et al., 2011b; Vertes et al., 2001). Initial recordings in anaesthetised animals found high levels of theta-responsive cells (i.e. cells that fire at a higher rate in the presence of theta) across all three thalamic subnuclei although only AV had strongly responsive cells, i.e. fired in bursts synchronous with theta; almost half of the cells recorded in AV demonstrated this activity (Vertes et al., 2001). Albo et al. (2006) also found theta-responsive cells across all nuclei of the ATN, up to 75 % of which were ‘theta-on’ cells showing theta phase-synchronous firing. Consistent with these animal studies, theta-responsive cells have also been recorded in human ATN (Sweeney-Reed et al., 2017, 2015; Sweeney-Reed et al., 2016). More recently, it has been established that both ‘true’ head direction cells (directional firing as a function of firing rate), as well as theta modulated head direction cells are present in AV (Tsanov et al., 2011a; Welday et al., 2011) and AM (Welday et al., 2011). In the latter study, Welday et al. (2011) also reported a population of neurons in AV and AM in which the frequency of theta cell burst firing was directionally modulated. In combination with speed-dependent theta modulated firing, such activity is theorised to be necessary for spatially-tuned firing (oscillatory interference model which will be discussed later; Burgess, 2008; Burgess et al., 2007). Consistently, the AM nucleus has been reported to contain a number of spatially tuned cells, including place cells and perimeter/border cells (Jankowski et al., 2015). Importantly, however, while it is known that the AD head direction signal is driven by lateral MB, it is not yet clear to what extent AV/AM theta-related directional activity is dependent on mammillothalamic inputs. ATN theta, however, is at least partly driven by the MBs, as procaine infusions into the MBs of anaesthetised rats reduces AV theta power (Zakowski et al., 2017). Furthermore, theta activity in AV is unaffected by dorsal hippocampal lesions, again suggesting that, similar to the supramammillary nucleus (Kirk and McNaughton, 1991), the MB-ATN pathway forms a medial diencephalic theta network that arises independently of the hippocampus (Talk et al., 2004).

## 4 A time and a place for the mammillothalamic pathway: a behavioural perspective

### 4.1 Temporal memory

#### 4.1.1 Patient studies

As mentioned in Section 1, much of our initial knowledge of medial diencephalic function has been drawn from patients with Korsakoff syndrome. This condition typically arises from thiamine deficiency but is often associated with chronic alcoholism. This neurological disorder is consistently linked to pathology within the medial MBs (Kopelman et al., 2009; Sullivan and Pfefferbaum, 2009) but it also causes widespread grey and white matter pathology as well as frontal pathology, often making it difficult to attribute specific cognitive impairments to specific brain regions. While Korsakoff syndrome is most commonly associated with a dense amnesic syndrome it can also cause numerous additional cognitive impairments including information processing and sensory deficits (Butters et al., 1975). Nevertheless, a consistent finding across studies involving Korsakoff syndrome patients, and indeed one of the earliest noted findings in this condition (Kessels and Kopelman, 2012; Korsakoff et al., 1996), is that patients are particularly impaired on temporal memory discrimination, i.e. the ability to remember the temporal order, or the temporal context in which events occur. It is this feature of Korsakoff syndrome that will be the focus of the current review.

In patients, anterograde temporal order memory is typically assessed in the laboratory using two types of tasks: within-list discrimination and/or between-list discrimination. Within-list discrimination involves a sample phase in which items (i.e. pictures or words) are presented sequentially followed by a test phase where the participant is required to decide which of two items was presented earlier in the sequence. Between-list discrimination involves the presentation of items in two distinct lists that are separated by a period of time; in the test phase the participant is required to determine the list from which the item originated. The between-list version is considered an easier task as there is a greater temporal separation between the test items and, therefore, more unique contextual cues. However, patients with Korsakoff syndrome are severely impaired on both versions, highlighting the severity of temporal memory impairment in this patient group (Hildebrandt et al., 2001; Hunkin et al., 2015; Huppert and Piercy, 1977; Kopelman et al., 1997; Meudell et al., 1985). The temporal memory impairments are not simply a reflection of the patients’ ability to remember the items, as they often perform normally when asked to decide which items had been presented, but are unable to put them into a temporal context.

As previously mentioned, one limitation when interpreting findings from Korsakoff syndrome studies is that the widespread grey and white matter damage makes it difficult to attribute specific memory impairments to specific brain regions. As such, there had been uncertainty as to whether the observed temporal memory impairments in this patient group resulted from medial diencephalic damage or from co-occurring frontal pathology; frontal pathology in patients has been repeatedly shown to affect strategic retrieval and the use of contextual cues. However, it has become evident that temporal memory impairments in Korsakoff syndrome patients can occur independently of frontal pathology. Firstly, Hunkin and Parkin (1993) found no correlation between Korsakoff syndrome patients’ performance on a temporal memory task and their performance on frontal tasks. Secondly, patients with selective medial diencephalic damage, targeting the MB region, also show very similar profiles to Korsakoff syndrome patients with marked impairments on temporal discrimination memory (Hildebrandt et al., 2001; Parkin and Hunkin, 1993). Furthermore, the findings from animal studies, where it is possible to assess the effects of even more selective damage, are also highly consistent with the MB-ATN pathway being particularly important for temporal memory.

#### 4.1.2 Animal models

Temporal memory tests in animals typically involve object or odour discrimination. Object recognition-based tasks capitalise on rats’ inherent preference for relative novelty, such that the amount of time spent exploring objects can indicate the extent to which animals remember the objects. Consistent with findings from patient studies, lesions of the ATN, MBs and MTT all leave intact the ability to discriminate novel from familiar items on tests of standard object recognition (Aggleton et al., 1995; Mitchell and Dalrymple-Alford, 2005; Moran and Dalrymple-Alford, 2003; Nelson and Vann, 2014; Warburton and Aggleton, 1999), making object discrimination a useful format to test temporal memory.

Several versions of temporal memory tasks have been employed in rodents, varying in their degree of similarity to human tasks. In the simplest version, rats distinguish between just two sets of items that are presented at distinct time points; intact animals should spend longer exploring objects presented first as they would be relatively more novel than the objects experienced more recently. This task is considerably less demanding than paradigms used to assess diencephalic lesion patients and, as such, it is perhaps unsurprising that neither ATN nor MTT lesions disrupt performance (Mitchell and Dalrymple-Alford, 2005; Nelson and Vann, 2017).

**Fig. 2:**
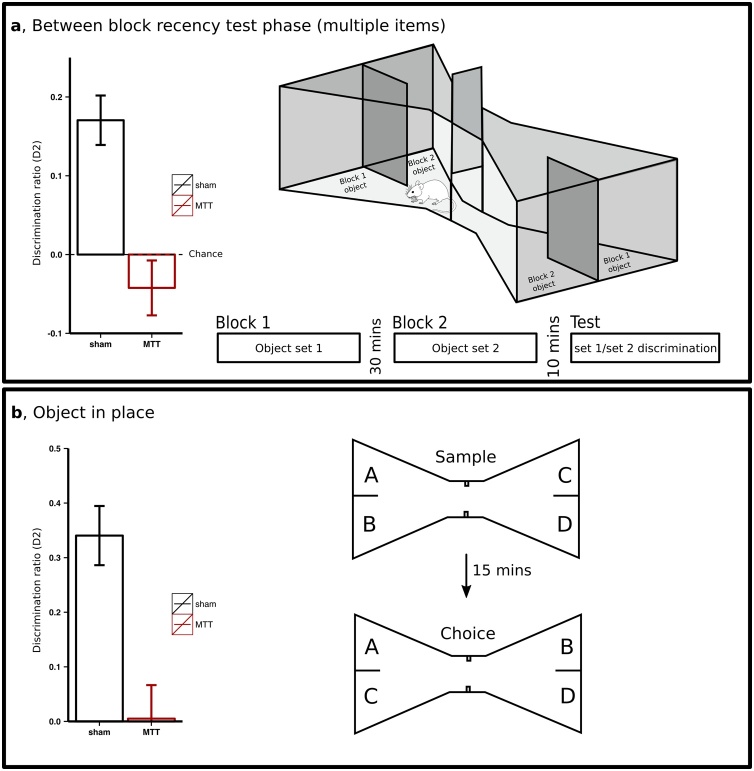
Mammillothalamic lesions (MTT) result in temporal (a) and contextual (b) memory impairments. In object recency experiments (a), rats are trained to shuttle from end-to-end in a bow tie maze. In each end of the maze, a partition separates the two objects which are positioned over a reward. On visits to each end of the maze, rats push away each object to retrieve the reward and are given the opportunity to explore each object. On the test sesion they are given a choice (“Test”) of one object from block 1, first encountered ∼40 min prior, or another object from block 2, encountered ∼10 min prior. Rats with MTT lesions are significantly impaired when required to discriminate between objects based on relative recency (Nelson and Vann, 2016). In object-in-place experiments (b), a sample phase consisting of one arrangement of four different objects (A, B, C and D), is followed, after a 15 min delay, by a choice phase in which objects B and C had switched positions. Both intramaze cues (i.e., cue cards) and extramaze cues are available. Rats with MTT lesions were significantly impaired in discriminating objects in a novel position (B and C) from those in a familiar position (A and D) (Nelson and Vann, 2014). In the both experiments, preferential exploration of the ‘novel’ object (i.e. less recent or displaced objects) was expressed as a discrimination ratio calculated as subtracting the time spent exploring the familiar from the novel item divided by the total time exploring both objects.

However, tasks exist that are more comparable to those used in patient studies (Hunkin et al., 2015) where animals are exposed to longer “lists” of sequentially-presented objects. Similar to the tasks used in patients, in the between-block discrimination, lists are presented in two distinct temporal blocks, while in the within-block discrimination, all objects are presented in a single continuous series (Dumont and Aggleton, 2013). Both versions of this task provide a measure of whether animals are able to discriminate objects based on their relative recency. However, as with the patient tasks, between-block discrimination is considered less demanding due to a greater temporal separation. Consistent with the findings from patients, both rats with MTT or ATN (including AD) lesions perform at chance levels on within-block recency tasks, however, in the case of the easier between-block recency task, only rats with MTT lesions were found to be impaired (and performing at chance levels; Fig. 2a) (Dumont and Aggleton, 2013; Nelson and Vann, 2017). This is somewhat surprising as one would expect ATN lesion effects to be at least as severe as those arising from MTT lesions given the MBs are thought to contribute to memory processes via their inputs to the ATN (Dillingham et al., 2015a). A likely explanation for this spared between-block recency performance in the ATN lesion animals is the relatively small lesion-size in this study as remaining tissue may have been sufficient to support the easier version of the task (Dumont and Aggleton, 2013).

Notably, ATN lesions (again, including AD) result in temporal memory impairments irrespective of the modality of the test stimuli or whether the tasks use spontaneous or rewarded behaviour. Wolff et al. (2006) rewarded rats for selecting one of two odours on the basis of presentation order during the sample phase. Rats with ATN lesions performed near chance on this task. Interestingly, performance was unaffected by the temporal spacing of the odours during the initial presentation (i.e. there was no effect of the number of intervening odours during the initial presentation). This was also found to be the case for object recognition tasks where rats withs rats with MTT or ATN lesions were unaffected by the degree of temporal separation (Dumont and Aggleton, 2013; Nelson and Vann, 2017), and it is also consistent with impairments on both within- and between-list tasks (Hunkin et al., 2015; Nelson and Vann, 2017) (but see Dumont and Aggleton, 2013).

The deficits observed with both between- and within-list tasks suggest the medial MB-ATN pathway is required for both fine-tuned temporal judgments and much coarser judgements over more temporally distinct periods. However, as mentioned earlier, neither MTT nor ATN lesions disrupt performance on the simplest version of the object recency task where animals discriminate between just two temporal events. This pattern of impairment is in contrast to frontal cortex lesions which result in an impairment on the simple temporal memory test (Warburton and Brown, 2015). Hunkin and Parkin (1993) suggested that frontal cortex and medial diencephalon both contribute to temporal memory processing but are necessary for different aspects. The frontal cortex may be necessary for the strategic recall of previously stored information whereas the medial diencephalon may support the encoding of temporal information (see also Huppert and Piercy, 1976, 1978).

In addition to assessingmemory for the temporal order of events, it is also possible to probe individuals' ability to estimate time intervals. Time estimation tasks have typically found impairments in patients with hippocampal damage (Kesner and Hopkins, 2001; Palombo et al., 2016). While there do not appear to be any studies that have assessed time estimation in humans with MB/ATN pathology, there are rodent studies that have assessed the contributions of the MBs to differential reinforcement of low-rate response tasks (DRL). This task requires animals to make a response in an operant chamber (e.g. lever-press) and then withhold a response for a specific period of time before making a further response to obtain a reward. Rats with MB lesions are severely impaired on this task (Smith and Schmaltz, 1979; Tonkiss and Rawlins, 1992). Impairments were found irrespective of whether animals were lesioned before or after training (Tonkiss and Rawlins, 1992), suggesting that they were not simply a result of difficutlties in learning task demands. Poor performance on this task can reflect either difficulties with response inhibition or with the perception of timing. However, there was no difference in overall response rate in the MB lesion animals (Smith and Schmaltz, 1979) and MB lesions do not typically affect response inhibition (Nelson and Vann, 2014), suggesting that the impairments observed on this task likely reflect an inability to accurately judge periods of time. These findings are similar to those from animals with hippocampal (Sinden et al., 1986) and entorhinal cortex lesions (Ramirez et al., 1995) both areas that have been linked to temporal processing. In contrast, subicular (Sinden et al., 1986) and postommissural fornix lesions (Johnson et al., 1977; Macdougall et al., 1969) appear to have little effect on performance, suggesting that the MBs’ involvement is at least partly independent of their hippocampal inputs (see also Tonkiss and Rawlins, 1992).

### 4.2 Contextual memory

#### 4.2.1 Patient studies

The importance of the MB-ATN axis for temporal processing has been clearly demonstrated across species, but it is possible that the temporal memory impairments form part of a more general difficulty in processing and encoding context. In this instance, context would be considered to be the environmental setting of the event or the background information (Kessels and Kopelman, 2012), i.e. the additional information that prevents the event being untethered in space and time.

While studies of Korsakoff syndrome patients report the most robust deficits on temporal memory tasks, additional evidence suggests further impairments in patients’ ability to access other details of memory, for example, source memory. Hildebrandt et al. (2001) tested a patient with MB pathology, resulting from a tumour, on a source memory task. Two experimenters each read a list of words and the patient’s task was to recall which word was read by which experimenter. The patient showed a clear impairment which contrasted with their intact performance when simply required to recognise previously presented words. Further studies have identified additional contextual memory impairments in Korsakoff patients. These include impaired spatial location judgements within a two-dimensional space, such as in object-location tasks (Kessels and Kopelman, 2012; Shoqeirat and Mayes, 1991). Performance on a more ethologically relevant task, where different word-pairs were presented at different times and at different spatial locations, was also affected in patients with Korsakoff syndrome (Pitel et al., 2008).

In a study by Tielemans et al. (2012), patients with Korsakoff syndrome and controls were presented with words paired with a photograph of a scene. During recall, the presentation of the photographs facilitated the memory for the words in the control group but not in the Korsakoff group. Again, this could reflect a role for the medial diencephalon in encoding or binding contextual information (Mayes and Downes, 1997; Tielemans et al., 2012). Consistent with deficient binding of complex features, Postma and colleagues found Korsakoff syndrome patients to be comparatively impaired on both object-location and temporal order tasks but much more severely impaired when faced with a combination of the two (Postma et al., 2006).

Since the deficits described above are features of Korsakoff syndrome, it remains possible they arise from more diffuse damage rather than specific damage to the medial-diencephalon. However, patients with thalamic infarcts that included damage encompassing the ATN and MTT were impaired when required to learn the correct path across a table-top maze (Stuss et al., 1988), while patient BJ (who had more discrete MB pathology as a result of an intranasal penetrating injury) was also impaired on an object-in-place task (Kapur, 1994). Furthermore, in patients with implanted electrodes, theta activity in the ATN was found to correlate with successful encoding of photographic scenes (Sweeney-Reed et al., 2015).

#### 4.2.2 Animal models

The pattern of contextual memory impairments, described above, is very similar across species. Item-place memory is disrupted in monkeys with either MB or ATN lesions (Croxson et al., 2012; Parker and Gaffan, 1997a, b). Similar object-location impairments have also been found in rodent models, as rats with MTT lesions are impaired on object-in-place tasks (Nelson and Vann, 2014), which require animals to combine the memory of an item with its spatial location within an arena or maze (Fig. 2b). These object-in-place impairments in rodents may reflect an inability to combine the object and place information but they could also reflect an impoverished representation of the spatial environment more generally. Rats with MTT and ATN lesions have difficulty discriminating between two corners of a room when tested on a go-no-go task where a pot in one location was rewarded whereas the pot in the other location was unrewarded (Dumont et al., 2014; Nelson and Vann, 2014). In the same way, MTT lesions impaired contextual discrimination in operant boxes where rats were required to learn one visuospatial context was rewarded while the other was unrewarded. In contrast, they had no difficulty on a similar task which required discrimination based on floor temperature (Vann et al., 2003) (but see Dumont et al., 2014). Likewise, MB lesions in mice and ATN lesions in rats disrupt contextual fear conditioning but spare cued fear conditioning (Celerier et al., 2004; Dupire et al., 2013; Marchand et al., 2014). Furthermore, re-exposure to a context that had previously been paired with a shock increased activity in medial MBs (Conejo et al., 2007). Taken together, the importance of the MBs and the ATN for contextual encoding is clearly evident across various tasks, yet, given the complex multisensory nature of these paradigms, the precise nature of the lesion-induced deficit awaits further clarification.

### 4.3 Impaired contextual encoding can result in greater mnemonic interference

Additional evidence suggests that poor contextual encoding can make it difficult to disambiguate familiar or similar items during recognition tasks. As described above, simple recognition tasks are often unaffected by damage to the MB-ATN pathway, however, this appears to be the case only when the task can be resolved by familiarity. Huppert and Piercy (1976) found Korsakoff patients to be impaired when required to make recognition judgments of high-frequency words but not low-frequency words. For the low-frequency words, it is possible to perform the task by simply using a familiarity judgment as the words are unlikely to have recently been encountered. However, greater exposure to high-frequency words will produce more interference. Therefore, to solve the task, participants need to specifically place the word within the context of the list. A somewhat analogous finding occurs in rats with MTT lesions where their ability to discriminate between new and previously presented objects became less accurate when they were presented with a greater number of objects. This effect was interpreted as a greater susceptibility of the lesioned animals to the increased interference between multiple objects with overlapping features (Nelson and Vann, 2017). The control group was unaffected by the increasing number of items suggesting they were more able to use additional cues, such as contextual cues, to help discriminate between these objects.

Likewise, the ATN appears particularly important for encoding contextual cues that can be used to reduce interference. In one study (Law and Smith, 2012), rats were trained to discriminate one set of odours for a reward in one context. They were then infused with either saline or muscimol into the ATN before being trained on a second discrimination where some of the rewarded odours from the first odour set were now unrewarded and vice versa. Half of the rats from each infusion group were trained in a novel context for the second odour-list and the remainder were trained in the same context. In saline infused (control) rats, there was a benefit of learning the second discrimination in a novel context, likely due to the reduced interference when able to use additional contextual cues. In contrast, animals that received muscimol infusions showed no benefit of the novel context, highlighting the importance of the ATN for encoding contextual cues that can be used to dissociate stimuli with high levels of interference (Law and Smith, 2012). Furthermore, the impairments observed following ATN inactivation were very similar to the impairments observed following hippocampal lesions (Butterly et al., 2012).

## 5 How might the mammillothalamic pathway contribute to contextual encoding?

**Fig. 3:**
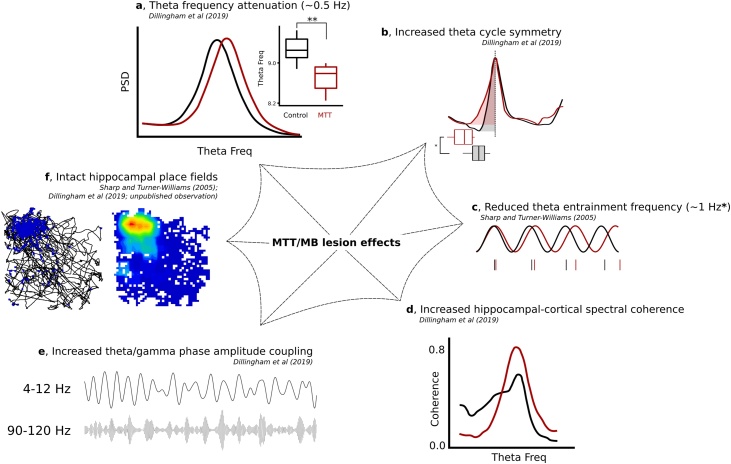
Physiological consequences of mammillothalamic tract (MTT) or mammillary body (MB) lesions. a, MTT lesions result in an attenuation of theta frequency in the CA1 subfield of the hippocampal formation and the retrosplenial cortex, the product of reduced ascending phase duration (trough-to-peak) of the theta cycle (b); c, lesions of MB (*and the supramammillary nucleus) reduce the frequency of hippocampal theta neuron entrainment by approximately 1 Hz; d, spectral coherence of hippocampal-retrospenial theta is increased in MTT lesioned animals, while theta-(high) gamma phase amplitude coupling in both regions is enhanced (e); f, Hippocampal place fields appear superficially intact following either MTT or MB lesions.

While the influence of the medial MB pathway on contextual and temporal processing has been well-established, comparably little is understood about the underlying physiological mechanisms that may be supporting this role. Based on current knowledge, the most straightforward explanation is that the medial MBs have a role in driving and/or modulating wider hippocampo-cortical circuits that are able to encode, generate and store contextual and temporal memories. As mentioned earlier (e.g. Section 3.1), theta oscillations are critical to a variety of mnemonic processes, providing an additional dimension within which neuronal firing dynamics represent experience, e.g. a spike-phase metric (Buzsáki, 2005). In light of what is known about the intrinsic electrophysiological properties of the medial MBs, as well as the theta-generating capacity of the VTg-medial MB axis (see Section 3), it is probable that deficits in contextual discrimination and temporal processing following experimental disconnection of mammillothalamic projections can be explained, at least in part, by disruption of theta-dependant mechanisms. In awake-behaving animals, disruption of MTT projections attenuates the frequency of theta band oscillations in the hippocampus and retrosplenial cortex (Dillingham et al., 2019) (Fig. 3a, c), but preserves rate coding for position (Sharp and Koester, 2008). Furthermore, inactivation of the ATN impairs grid cell periodicity in the entorhinal cortex (Winter et al., 2015). Under anaesthesia, both MB and ATN inactivation attenuate hippocampal theta and MB inactivation reduces theta power in AV (Zakowski et al., 2017), highlighting the importance on ascending MB projections for theta-related firing across memory networks.

**Fig. 4:**
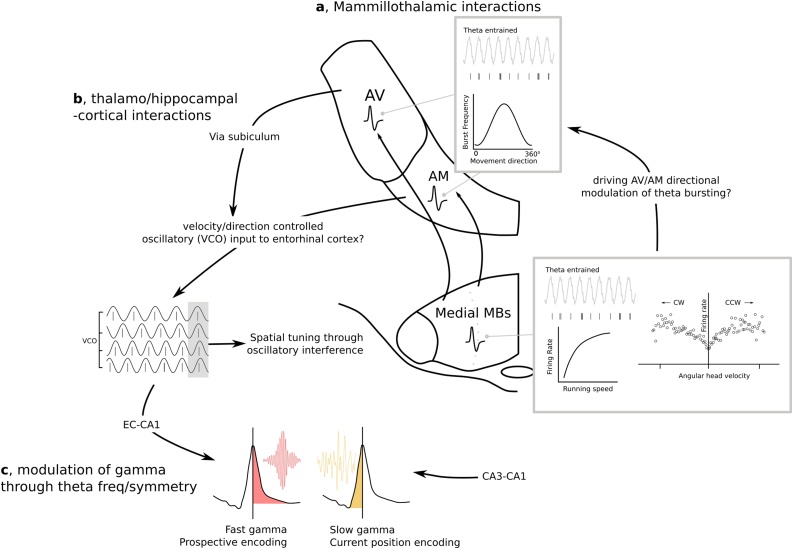
A possible route through which the medial mammillary bodies (MBs) and their projections to the anterior thalamic nuclei might influence contextual representations in the hippocampal formation. a, As well as head direction cells, anteroventral (AV) and anteromedial (AM) thalamic nuclei contain a high proportion of theta entrained neurons, a proportion of which encode direction through through the frequency of theta bursting (Welday et al., 2011). The medial MBs also house a high proportion of theta entrained neurons as well as those that are correlated with running speed, and angular head velocity. If it is the case that AM/AV activity is driven by the MBs, ascending MB activity may influence parahippocampal periodic tuning through modulation of thalamic directionally/velocity controlled oscillators; activity which is critical to oscillatory interference models of entorhinal (EC) grid cell periodicity (b; see Winter et al., 2015); Direct (AM-EC) and indirect (AV-subiculum-EC) thalamic influence over the EC may in turn modulate theta frequency/cycle asymmetry (Dillingham et al., 2019) and the related fast gamma low gamma ratio (which are dominant in the descending and ascending phase of the theta cycle, respectively) in CA1. The fast gamma (EC-CA1 derived), slow gamma (CA3-CA1 derived) ratio is thought to reflect a variable focus on prospective (future action) planning (fast gamma dominant) vs. current position encoding (slow gamma dominant).

Contextual and temporal memory processes are multi-faceted and are therefore likely undepinned by numerous complementary mechanisms. As such, selective disruption to different but parallel processing streams involved in generation and maintenance of contextual/temporal memory may still result in qualitatively equivalent deficits. In other words, insufficient contextual detail at any stage of mnemonic processing may outwardly manifest in a similar fashion. As suggested above, the MB-ATN axis may be specifically tuned (via theta oscillations) to process and relay context-rich and time-critical information that is further integrated and distributed to higher-order areas by thalamocortical circuits. As will be outlined below, mounting experimental evidence indeed points toward a widespread role of the MBs in supporting hippocampo-cortical functioning thus highlighting the potential complexity of mechanisms through which the MBs may affect contextual and temporal processing. Consequently, in the sections that follow, we consider how the medial MB-ATN axis may contribute to hippocampo-cortical mechanisms which are thought to underpin the formation of spatio-temporal representations (Fig. 4).

### 5.1 Theta-sequences

Despite MB/MTT lesions disrupting spatial memory and attenuating hippocampal theta frequency, they appear to leave place cell firing in CA1 largely intact (Sharp and Koester, 2008; unpublished findings from our lab) (Fig. 3f). A similar dissociation between effects on theta and place cell firing is also found following medial septal inactivation (Brandon et al., 2014). On one level, this suggests that CA1 place cell activity in and of itself is not sufficient to support spatial encoding. However, it appears likely that simply measuring place cell firing is likely to overlook many additional complexities related to temporal and contextual processing. Hippocampal place cells discharge as a function of the position of the animal in its environment (O’Keefe and Dostrovsky, 1971). In this context, theta oscillations (4–12 Hz) provide a temporal framework within which the resolution of the spatial representation is enhanced. So-called theta precession (O’Keefe and Recce, 1993) is a mechanism by which the position of an animal within a place field is encoded by the phase, within a theta cycle, at which action potentials take place; firing occurs at progressively earlier phases (from the peak, towards the theta trough) of subsequent theta cycles. Time-compressed sequences of past and future locations are represented by assemblies of phase-precessing neurons (Dragoi and Buzsaki, 2006; Huxter et al., 2003). Such theta sequences develop within a novel environment over the course of repeated experience to the same trajectory (Feng et al., 2015), possibly in alignment with experience/familiarity-dependent asymmetric expansion of place fields (Mehta et al., 1997, 2000). The interdependence of phase precession and theta cycles is not fully understood (Drieu and Zugaro, 2019), however, it is generally accepted that it constitutes an important mechanism that enables associated representations to be linked together, thus providing spatio-temporal information about an event. Many characteristics of theta oscillations and associated spiking activity are modulated by ambulatory activity, e.g. exploration and rearing; at higher running speeds, theta frequency increases (Hinman et al., 2011; Jeewajee et al., 2008; Vanderwolf, 1969) and the ‘slope’ of phase precession within a theta cycle becomes steeper such that the accuracy of the positional representation is maintained in spite of the increase in the rate of information flow (Geisler et al., 2007; McNaughton et al., 1983). In the hippocampus, increases in theta frequency are primarily the result of changes in the ascending (trough-to-peak) phase duration of the theta cycle. While running speed-dependent changes in ascending phase duration are not altered by MTT lesions, overall durations of ascending phases in theta cycles are greater (Dillingham et al., 2019) (Fig. 3b). A potential consequence of this shift in oscillatory dynamics might be that phase precession is modified e.g., through a change in the slope of phase precession of pyramidal cell/interneurons, and/or a change in the rate of change in speed-dependent firing. In both instances, the rate, as well as the resolution, at which positional information is updated would likely be reduced, thus impacting spatio-temporal processing (but see Carpenter et al., 2017).

### 5.2 Hippocampal time cells

In addition to simply representing positional space, hippocampal pyramidal neurons can also encode further aspects of contextual experience: firing may preferentially reflect the direction in which a place field is entered (O’Keefe and Dostrovsky, 1971), can be modulated by linear velocity (Gois and Tort, 2018; Hirase et al., 1999; Maurer et al., 2005; McNaughton et al., 1983; Wiener et al., 1989), and also has the capacity to encode temporal information (Mau et al., 2018; Pastalkova et al., 2008). Time-dependent activity in CA1 appears dependent on the demands of the task or activity in which the animal is engaged (MacDonald et al., 2011). In this respect, periodic firing of pyramidal neurons is representative of specific temporal features, i.e. the “when” aspect of episodic memory. Hippocampal time cells may not explicitly encode time but instead a combination of time in association with place, direction, and/or speed or reward (Muller et al., 1994; Poucet and Hok, 2017). It has been suggested that the variable temporal framework within which these partially decorrelated place cells represent time windows, or features, are modulated by inputs from the lateral entorhinal cortex, within which task- and spatially-*independent* representations of time have been described (Tsao et al., 2018). Entorhinal time cells exhibit cycles of low firing rate ramping to higher frequency firing over the course of relatively long time periods. In turn, this dynamic ramping activity is thought to be transformed to a discrete hippocampal time cell code (Rolls and Mills, 2019). This additional temporal information enables discrete spatial locations to be linked within theta sequences and also provides a mechanism for encoding distance travelled (Kraus et al., 2013).

Several computational models have been proposed to characterize potential mechanisms by which spatially tuned firing is generated. Attractor network (Samsonovich and McNaughton, 1997) and oscillatory interference models (Burgess et al., 2007) are two popular examples. While the former provides a mechanism based on neuronal firing alone, of more relevance to the current discussion (relating to the medial MBs as a source of speed and potentially head direction modulated theta), oscillatory interference models provide a mechanism through which spike timing dynamics interact with multiple oscillatory sources to generate spatial periodicity. Oscillatory interference models posit that spatial tuning of neuronal firing is generated by the phase interference of speed- and velocity-dependent oscillators (Burgess, 2008; Burgess et al., 2007). Entorhinal speed cells discharge purely as a function of running speed and while the inputs driving this modulation are unknown, they are thought to be independent of septal activity (Carvalho et al., 2020; Dannenberg et al., 2019). Given the acute effects of ATN inactivation on entorhinal activity (Winter et al., 2015), an open question is whether or not indirect MB-entorhinal inputs (via ATN or subicular cortices; Nassar et al., 2018) influence the entorhinal-hippocampal axis. Idiothetic input from the MB-ATN axis (e.g. velocity-dependent theta frequency, speed cells) could be important for those hippocampal time cells that represent a ‘distance-travelled over time’ metric, rather than a specific temporal feature of a task (Kraus et al., 2013). Thus, in parallel with the likely influence of the lateral MB on the entorhinal head direction signal (Winter et al., 2015) medial MB speed-modulated cells (Fig. 4a) and associated speed-dependent VTg-MB theta may represent an important component of path integration related information, i.e. a velocity controlled oscillatory source which contributes to spatial periodic firing but also, potentially, time cell representations of distance travelled. This would be consistent with impairments in dead reackoning observed in rats with MTT lesions (Winter et al., 2011).

### 5.3 Gamma oscillations

Hippocampal gamma oscillations (30−140 Hz) are phase modulated by theta (Belluscio et al., 2012; Colgin, 2015b; Colgin et al., 2009; Lasztoczi and Klausberger, 2016) and, on a cellular level, gamma-theta interactions are thought to be important in the temporal orchestration of place cell ensemble activation (Fernandez-Ruiz et al., 2017; Schomburg et al., 2014; Senior et al., 2008). Gamma oscillations are typically subdivided into slow (30−50 Hz), fast (50−90 Hz) and epsilon (90−140 Hz) bands. The ratio of low/high gamma modulation is thought to reflect a distinction between the representation of current location (high gamma) versus that of the future trajectory (prospective encoding; low gamma) (Bieri et al., 2014; Hasselmo et al., 2002; Zheng et al., 2016). The former is thought to be predominantly reliant on entorhinal-CA1 projections, as opposed to the latter, where CA3-CA1 projections dominate (Brun et al., 2002; Colgin, 2015b) (Fig. 4b). In contrast to a typical sinusoidal representation of filtered theta oscillations, theta cycles are typically asymmetrical, with a shorter ascending phase and a longer descending phase (Amemiya and Redish, 2018; Belluscio et al., 2012; Buzsaki et al., 1985). Low gamma is dominant in more symmetrical theta cycles whereas high gamma is dominant in more asymmetrical cycles, reflecting the fact that high gamma is entrained to the descending slope of the cycle, while low gamma is embedded within the ascending phase (Belluscio et al., 2012; Colgin, 2015a) (Fig. 4b). Associated with a reduction in theta frequency, lesions of the MTT result in more symmetrical hippocampal and cortical (retrosplenial) theta cycles (Dillingham et al., 2019) (Fig. 3b). Following MTT lesions, hippocampal theta cycles exhibited increased ascending phase durations, potentially reflecting an increased low-high gamma ratio, which would reduce the capacity to encode current location. The lesions also resulted in changes to the theta cycle in retrosplenial cortex: descending phase cycle durations were significantly increased, coupled with an increase in the peak frequency and theta phase amplitude coupling of high/epsilon gamma (Dillingham et al., 2019) (Fig. 4e). Together, these phenomena may reflect a reorganisation in the dominance of CA3 versus entorhinal hippocampal inputs which might reflect compromised contextual encoding (Alexander and Nitz, 2015; Mao et al., 2017; Miller et al., 2019).

### 5.4 Place field remapping

A consistent finding following disruption of major afferent hippocampal pathways (e.g. septal or entorhinal) is an increase in the frequency of place field remapping within familiar environments. Inactivation of the medial septum abolishes theta in the hippocampus and induces remapping of CA1 place cells (although place fields retain the spatial content and stability once remapped; Brandon et al., 2014). Upon recovery from temporary septal inactivation, place cells were found to recover their previous spatial organisation. Similarly, lesions of the medial entorhinal cortex induce remapping of CA1 place fields (Miao et al., 2015) but without impairing spatial information content or field stability. Chemogenetic inactivation of the medial entorhinal cortex, however, results in a strong reduction in phase precession during exploration and a reduction in the efficacy of replay of past exploratory trajectories (Kanter et al., 2017). A similar pattern of effects is found following systemic administration of the NMDA antagonist CPP, which also leaves place fields intact but disrupts the replay of recent events (Kentros et al., 1998; Rowland et al., 2011). In spite of place fields being intact following CPP administration, replay of trajectories experienced during CPP treatment was abolished and replay of experiences prior to CPP administration was observed instead. In these instances, spatial encoding appeared intact at a superficial level, however, mechanisms underlying the experiential transition from novel to familiar environment were impaired. Given the partial overlap in the effects of septal, entorhinal and medial diencephalic lesions, it is likely that, in spite of the seemingly intact CA1 place cell firing, animals with MTT lesions still experience impoverished contextual encoding that could contribute to the behavioural impairments observed.

### 5.5 Neurogenesis: differentiating memories through context

New neurons are continuously generated in the subventricular zone and granule cell layer of the dentate gyrus. These adult-generated dentate granule cells are thought to contribute to hippocampal-dependent memory (Deng et al., 2010; Shors, 2008). The integration of newborn cells into the dentate gyrus has been specifically linked to contextual learning and to reducing interference across overlapping memories (Miller and Sahay, 2019; Winocur et al., 2012). For example, Winocur et al. (2012) suggested that newborn dentate gyrus cells were particularly important for separating target memories from those with overlapping contextual features. One proposal is that hippocampal neurogenesis enables novel events to be encoded into newly-born populations of cells, leaving older neurons to represent older memories; this representation of events by distinct neuronal clusters provides a mechanism that reduces interference across events. A further suggestion is that new populations of neurons act as a time-stamp as events separated by time will be encoded by different populations of new neurons within the dentate gyrus. Immature hippocampal cells have been shown to be particularly important for spatial encoding and for novel learning (Tronel et al., 2015; Vukovic et al., 2013) and, as such, many of the processes linked to neurogenesis appear to closely match those processes supported by the medial diencephalon.

Consistent with this observation, animal models of Korsakoff syndrome show reduced hippocampal neurogenesis (Vetreno et al., 2011; Zhao et al., 2008). Again, the use of thiamine-deficient models makes it difficult to determine whether this effect was specifically driven by medial diencephalic damage. However, our recent findings suggest this reduction in neurogenesis may specifically reflect damage along the medial MB-ATN axis as a reduction in neurogenesis was found following selective lesions to the MTT. Not only were there fewer newborn cells in the dentate gyrus, as measured by doublecortin, but the cells that were present were less complex with fewer dendritic branches (Dillingham et al., 2019). There is also evidence that stimulation of the ATN promotes hippocampal neurogenesis, as does stimulation of the entorhinal cortex (Encinas et al., 2011; Stone et al., 2011; Toda et al., 2008). The mammillothalamic axis may, therefore, support aspects of contextual encoding via its distal effects on neurogenesis. However, any disruption to neurogenesis as a result of medial diencephalic pathology is likely to only partially account for the memory effects observed as the number of newborn cells was only moderately reduced (by 30 %) in the MTT-lesion model (Dillingham et al., 2019), whereas more pronounced spatial memory deficits are typically reported after a more complete loss of adult newborn cells (e.g., Jessberger et al., 2009). Furthermore, the time-frame of neurogenesis is unlikely to provide a timing mechanism for short term temporal discrimination but it may help to make temporally distal memories more distinct.

### 5.6 Retrosplenial cortex dysfunction

In addition to its contribution to hippocampal function, the MB-ATN pathway may further support temporal and contextual processing via its dense connections with the retrosplenial cortex. The retrosplenial cortex has been consistently linked to spatial memory and navigation in both rodents and humans (Milczarek and Vann, 2020; Vann et al., 2009a), supported by recordings of spatially-related neural responses (Alexander and Nitz, 2015; Mao et al., 2018; Milczarek et al., 2018; Miller et al., 2019). Furthermore, many of the spatio-temporal impairments observed in rodents with MTT/ATN lesions (see Section 4) are also found following retrosplenial lesions, including contextual fear conditioning (Keene and Bucci, 2008), place discrimination (Hindley et al., 2014b), and temporal discrimination (Powell et al., 2017). Loss of the ascending inputs from the MB-ATN pathway has been repeatedly shown to disrupt normal retrosplenial function, as measured by multiple activity markers. For example, lesions of the MTT and/or ATN (and to a lesser extent lateral MBs) produce a striking reduction in immediate-early gene expression in the retrosplenial cortex (Frizzati et al., 2016; Jenkins et al., 2004; Vann, 2018; Vann and Albasser, 2009), as well as reduce long-term depression (Garden et al., 2009). Moreover, retrosplenial hypometabolism has been reported in Korsakoff syndrome patients (Reed et al., 2003). Our recent study in MTT lesioned rats showed not only disrupted retrosplenial theta rhythm, but also an increase in hippocampal-retrosplenial coherence (Fig. 3d), suggesting a loss of information content due to excessive synchronisation. Furthermore, using MR imaging in rats, we demonstrated a relative reduction in a measure of experience-driven grey matter diffusivity (elicited by spatial training the the radial-arm maze) in MTT-lesioned rats compared to controls (Dillingham et al., 2019). As such, the medial MB-ATN pathway is likely to provide critical input for temporal and contextual processing within the retrosplenial cortex.

## 6 Conclusions

The MB-ATN pathway has consistently been found to be critical for both temporal and contextual discrimination across species. There are many overlapping features in the mechanisms underlying temporal and contextual processing and considering time as an additional dimension of space could explain the similarity in impairments involving both features. Context provides a clear additional means for segmenting periods of time, with changing contexts likely providing mnemonic boundaries for discrete epochs.

While the importance of the MBs and ATN for contextual memory has been repeatedly demonstrated, there are still many outstanding questions in terms of what these impairments reflect. Is it impoverished contextual encoding? an impairment in combining features? or perhaps both? The behavioural tests typically used in animals require integrated learning, e.g. combining reward, shock, an object, or an odour with context, making it difficult to tease apart the underlying impairment. Resolving this issue may depend on electrophysiological recordings, rather than behaviour alone, which would enable context-related neural signatures to be assessed in intact animals and following the disruption of medial MB-ATN connections. As described above, on a superficial level, hippocampal place fields appear intact following MTT lesions, which might suggest that behavioural impairments are not due to failure at encoding space/context but instead an inability to integrate features within these contexts. It is clear, however, that place fields alone remain a relatively crude measure of spatial representation. Consequently, a more comprehensive understanding of how the medial MB-ATN pathway supports the encoding of theta sequences and place cell/oscillatory interactions is needed in order to make a clearer determination of their role in contextual encoding and processing.

Despite increasing evidence that medial MB-ATN pathway is required for normal hippocampal neural activity, the anatomical pathway(s) that supports these mechanisms is still unknown. There are multiple direct and indirect routes into the hippocampal/parahippocampal regions from both the AV and AM subnuclei. It is, therefore, a clear goal to elucidate the functional importance of each pathway to fully map out the neuroanatomical substrates supporting temporal and contextual memory. In the same way, there are multiple mechanisms via which the medial MB-ATN pathway may be supporting temporal-contextual memory, across both the hippocampus and retrosplenial cortex, and a critical future aim is to identify which mechanisms support which aspects of behaviour.

The current review has predominantly focused on the role of the medial MB pathway in contextual learning. Nevertheless, the possibility that the lateral MB pathway may be providing complementary information required for certain aspects of contextual learning cannot be overlooked. Lesions of the lateral MBs, for example, have been found to disrupt the use of geometric and allocentric cues in spatial learning and navigation (Vann, 2005, 2011) while directional firing in the lateral MB network has been shown to be anchored to local spatial environments, e.g. in the T-maze during alternation learning (Dudchenko et al., 2005). Relatedly, lesions of the lateral MBs abolish the directional specificity of hippocampal place field repetition, potentially resulting in increased overlap across spatial representations and, thus, a reduced capacity to discriminate different spatial contexts (Harland et al., 2017). Together, these examples highlight the importance of teasing apart these two parallel streams when attempting to understand the functional role of the MB-ATN pathway. Although certain similar behavioural consequences may be observed following disruption to each of these pathways, the underlying mechanisms are likely to be very different.

While MB-ATN pathology has been traditionally linked to Korsakoff syndrome, there is increasing evidence for MB-ATN pathology across a wide range of neurological disorders that are associated with memory impairments, e.g. neonatal hypoxia (Molavi et al., 2019) and other conditions associated with low oxygen levels (Cabrera-Mino et al., 2020), Down syndrome (Perry et al., 2019) and colloid cysts (Denby et al., 2009; Vann et al., 2009b). Together, these various conditions highlight the pressing need to better understand the mechanisms by which the MBs and ATN support normal memory in order to ultimately identify routes to restoring function.

## Funding

All authors are funded by a Wellcome Trust Senior Research Fellowship awarded to SDV (WT 212273/Z/18/Z).

## References

[bib0005] Aggleton J.P., Neave N., Nagle S., Hunt P.R. (1995). A comparison of the effects of anterior thalamic, mamillary body and fornix lesions on reinforced spatial alternation. Behav. Brain Res..

[bib0010] Albo Z., Viana Di Prisco G., Vertes R. (2006). Anterior thalamic unit discharge profiles and coherence with hippocampal theta rhythm. Thalamus Relat. Syst..

[bib0015] Alexander A.S., Nitz D.A. (2015). Retrosplenial cortex maps the conjunction of internal and external spaces. Nat. Neurosci..

[bib0020] Allen G.V., Hopkins D.A. (1989). Mamillary body in the rat: topography and synaptology of projections from the subicular complex, prefrontal cortex, and midbrain tegmentum. J. Comp. Neurol..

[bib0025] Alonso A., Llinas R.R. (1992). Electrophysiology of the mammillary complex in vitro. II. Medial mammillary neurons. J. Neurophysiol..

[bib0030] Alvarez-Bolado G., Zhou X., Voss A.K., Thomas T., Gruss P. (2000). Winged helix transcription factor Foxb1 is essential for access of mammillothalamic axons to the thalamus. Development.

[bib0035] Amemiya S., Redish A.D. (2018). Hippocampal theta-gamma coupling reflects state-dependent information processing in decision making. Cell Rep..

[bib0040] Amilhon B., Huh C.Y., Manseau F., Ducharme G., Nichol H., Adamantidis A., Williams S. (2015). Parvalbumin interneurons of hippocampus tune population activity at theta frequency. Neuron.

[bib0045] Babmindra V.P., Grachev I.I., Chernysheva M.P., Pavlenko I.N., Karyakin M.G., Petrov Y.A. (1979). Correlation between morphology and function in the rat hippocampus and hypothalamus. Neirofiziologiya.

[bib0050] Bassant M.H., Poindessous-Jazat F. (2001). Ventral tegmental nucleus of Gudden: a pontine hippocampal theta generator?. Hippocampus.

[bib0055] Belluscio M.A., Mizuseki K., Schmidt R., Kempter R., Buzsaki G. (2012). Cross-frequency phase-phase coupling between theta and gamma oscillations in the hippocampus. J. Neurosci..

[bib0060] Bernstein H.G., Krause S., Krell D., Dobrowolny H., Wolter M., Stauch R., Ranft K., Danos P., Jirikowski G.F., Bogerts B. (2007). Strongly reduced number of parvalbumin-immunoreactive projection neurons in the mammillary bodies in schizophrenia: further evidence for limbic neuropathology. Ann. N. Y. Acad. Sci..

[bib0065] Bieri Kevin W., Bobbitt Katelyn N., Colgin Laura L. (2014). Slow and fast gamma rhythms coordinate different spatial coding modes in hippocampal place cells. Neuron.

[bib0070] Blair H.T., Cho J.W., Sharp P.E. (1998). Role of the lateral mammillary nucleus in the rat head direction circuit: a combined single unit recording and lesion study. Neuron.

[bib0075] Brandon M.P., Koenig J., Leutgeb J.K., Leutgeb S. (2014). New and distinct hippocampal place codes are generated in a new environment during septal inactivation. Neuron.

[bib0080] Brun V.H., Otnæss M.K., Molden S., Steffenach H.-A., Witter M.P., Moser M.-B., Moser E.I. (2002). Place cells and place recognition maintained by direct entorhinal-hippocampal circuitry. Science.

[bib0085] Burgess N. (2008). Grid cells and theta as oscillatory interference: theory and predictions. Hippocampus.

[bib0090] Burgess N., Barry C., O’Keefe J. (2007). An oscillatory interference model of grid cell firing. Hippocampus.

[bib0095] Butterly D.A., Petroccione M.A., Smith D.M. (2012). Hippocampal context processing is critical for interference free recall of odor memories in rats. Hippocampus.

[bib0100] Butters N., Cermak L.S., Jones B., Glosser G., Gross M.M. (1975). Some analyses of the information processing and sensory capacities of alcoholic korsakoff patients. Alcohol Intoxication and Withdrawal: Experimental Studies II.

[bib0105] Buzsáki G. (2005). Theta rhythm of navigation: link between path integration and landmark navigation, episodic and semantic memory. Hippocampus.

[bib0110] Buzsaki G., Rappelsberger P., Kellenyi L. (1985). Depth profiles of hippocampal rhythmic slow activity (’theta rhythm’) depend on behaviour. Electroencephalogr. Clin. Neurophysiol..

[bib0115] Byatt G., Dalrymple-Alford J.C. (1996). Both anteromedial and anteroventral thalamic lesions impair radial-maze learning in rats. Behav. Neurosci..

[bib0120] Cabrera-Mino C., Roy B., Woo M.A., Singh S., Moye S., Halnon N.J., Lewis A.B., Kumar R., Pike N.A. (2020). Reduced brain mammillary body volumes and memory deficits in adolescents who have undergone the Fontan procedure. Pediatr. Res..

[bib0125] Carlesimo G.A., Serra L., Fadda L., Cherubini A., Bozzali M., Caltagirone C. (2007). Bilateral damage to the mammillo-thalamic tract impairs recollection but not familiarity in the recognition process: a single case investigation. Neuropsychologia.

[bib0130] Carpenter F., Burgess N., Barry C. (2017). Modulating medial septal cholinergic activity reduces medial entorhinal theta frequency without affecting speed or grid coding. Sci. Rep..

[bib0135] Carvalho M.M., Tanke N., Kropff E., Witter M.P., Moser M.B., Moser E.I. (2020). A brainstem locomotor circuit drives the activity of speed cells in the medial entorhinal cortex. Cell Rep..

[bib0140] Casatti C.A., Elias C.F., Sita L.V., Frigo L., Furlani V.C., Bauer J.A., Bittencourt J.C. (2002). Distribution of melanin-concentrating hormone neurons projecting to the medial mammillary nucleus. Neuroscience.

[bib0145] Celerier A., Pierard C., Beracochea D. (2004). Effects of ibotenic acid lesions of the dorsal hippocampus on contextual fear conditioning in mice: comparison with mammillary body lesions. Behav. Brain Res..

[bib0150] Celio M.R. (1990). Calbindin D-28k and parvalbumin in the rat nervous system. Neuroscience.

[bib0155] Cembrowski M.S., Phillips M.G., DiLisio S.F., Shields B.C., Winnubst J., Chandrashekar J., Bas E., Spruston N. (2018). Dissociable structural and functional hippocampal outputs via distinct subiculum cell classes. Cell.

[bib0160] Chalfonte B.L., Verfaellie M., Johnson M.K., Reiss L. (1996). Spatial location memory in amnesia: binding item and location information under incidental and intentional encoding conditions. Memory.

[bib0165] Cipolotti L., Husain M., Crinion J., Bird C.M., Khan S.S., Losseff N., Howard R.S., Leff A.P. (2008). The role of the thalamus in amnesia: a tractography, high-resolution MRI and neuropsychological study. Neuropsychologia.

[bib0170] Clark B.J., Bassett J.P., Wang S.S., Taube J.S. (2010). Impaired head direction cell representation in the anterodorsal thalamus after lesions of the retrosplenial cortex. J. Neurosci..

[bib0175] Colgin L.L. (2013). Mechanisms and functions of theta rhythms. Annu. Rev. Neurosci..

[bib0180] Colgin L.L. (2015). Do slow and fast gamma rhythms correspond to distinct functional states in the hippocampal network?. Brain Res..

[bib0185] Colgin L.L. (2015). Theta-gamma coupling in the entorhinal-hippocampal system. Curr. Opin. Neurobiol..

[bib0190] Colgin L.L., Denninger T., Fyhn M., Hafting T., Bonnevie T., Jensen O., Moser M.B., Moser E.I. (2009). Frequency of gamma oscillations routes flow of information in the hippocampus. Nature.

[bib0195] Conejo N.M., Gonzalez-Pardo H., Lopez M., Cantora R., Arias J.L. (2007). Induction of c-Fos expression in the mammillary bodies, anterior thalamus and dorsal hippocampus after fear conditioning. Brain Res. Bull..

[bib0200] Croxson P.L., Browning P.G., Gaffan D., Baxter M.G. (2012). Acetylcholine facilitates recovery of episodic memory after brain damage. J. Neurosci..

[bib0205] Dannenberg H., Kelley C., Hoyland A., Monaghan C.K., Hasselmo M.E. (2019). The firing rate speed code of entorhinal speed cells differs across behaviorally relevant time scales and does not depend on medial septum inputs. J. Neurosci..

[bib0210] de Lima M.A., Baldo M.V., Canteras N.S. (2017). A role for the anteromedial thalamic nucleus in the acquisition of contextual fear memory to predatory threats. Brain Struct. Funct..

[bib0215] Denby C.E., Vann S.D., Tsivilis D., Aggleton J.P., Montaldi D., Roberts N., Mayes A.R. (2009). The frequency and extent of mammillary body atrophy associated with surgical removal of a colloid cyst. AJNR Am. J. Neuroradiol..

[bib0220] Deng W., Aimone J.B., Gage F.H. (2010). New neurons and new memories: how does adult hippocampal neurogenesis affect learning and memory?. Nat. Rev. Neurosci..

[bib0225] Dillingham C.M., Vann S.D. (2019). Why isn’t the head direction system necessary for direction? Lessons from the lateral mammillary nuclei. Front. Neural Circuits.

[bib0230] Dillingham C.M., Frizzati A., Nelson A.J., Vann S.D. (2015). How do mammillary body inputs contribute to anterior thalamic function?. Neurosci. Biobehav. Rev..

[bib0235] Dillingham C.M., Holmes J.D., Wright N.F., Erichsen J.T., Aggleton J.P., Vann S.D. (2015). Calcium-binding protein immunoreactivity in Gudden’s tegmental nuclei and the hippocampal formation: differential co-localization in neurons projecting to the mammillary bodies. Front. Neuroanat..

[bib0240] Dillingham C.M., Milczarek M.M., Perry J.C., Frost B.E., Parker G.D., Assaf Y., Sengpiel F., O’Mara S.M., Vann S.D. (2019). Mammillothalamic disconnection alters hippocampocortical oscillatory activity and microstructure: implications for diencephalic amnesia. J. Neurosci..

[bib0245] Donovan M.K., Wyss J.M. (1983). Evidence for some collateralization between cortical and diencephalic efferent axons of the rat subicular cortex. Brain Res..

[bib0250] Dragoi G., Buzsaki G. (2006). Temporal encoding of place sequences by hippocampal cell assemblies. Neuron.

[bib0255] Drieu C., Zugaro M. (2019). Hippocampal sequences during exploration: mechanisms and functions. Front. Cell. Neurosci..

[bib0260] Dudchenko P.A., Muir G.M., Frohardt R.J., Taube J.S., Wiener S.I., Taube J.S. (2005). What does the Head Direction Cell System Actually do. Head Direction Cells and the Neural Mechanisms of Spatial Orientation.

[bib0265] Dudchenko P.A., Wood E.R., Smith A. (2019). A new perspective on the head direction cell system and spatial behavior. Neurosci. Biobehav. Rev ..

[bib0270] Dumont J.R., Aggleton J.P. (2013). Dissociation of recognition and recency memory judgments after anterior thalamic nuclei lesions in rats. Behav. Neurosci..

[bib0275] Dumont J.R., Amin E., Aggleton J.P. (2014). Selective importance of the rat anterior thalamic nuclei for configural learning involving distal spatial cues. Eur. J. Neurosci..

[bib0280] Dupire A., Kant P., Mons N., Marchand A.R., Coutureau E., Dalrymple-Alford J., Wolff M. (2013). A role for anterior thalamic nuclei in affective cognition: interaction with environmental conditions. Hippocampus.

[bib0285] Encinas J.M., Hamani C., Lozano A.M., Enikolopov G. (2011). Neurogenic hippocampal targets of deep brain stimulation. J. Comp. Neurol..

[bib0290] Feng T., Silva D., Foster D.J. (2015). Dissociation between the experience-dependent development of hippocampal theta sequences and single-trial phase precession. J. Neurosci..

[bib0295] Fernandez-Ruiz A., Oliva A., Nagy G.A., Maurer A.P., Berenyi A., Buzsaki G. (2017). Entorhinal-CA3 dual-input control of spike timing in the hippocampus by theta-gamma coupling. Neuron.

[bib0300] Field T.D., Rosenstock J., King E.C., Greene E. (1978). Behavioral role of the mammillary efferent system. Brain Res. Bull..

[bib0305] Fortin M., Parent A. (1997). Distribution of calretinin, calbindin-D28k and parvalbumin in the hypothalamus of the squirrel monkey. J. Chem. Neuroanat..

[bib0310] Frizzati A., Milczarek M.M., Sengpiel F., Thomas K.L., Dillingham C.M., Vann S.D. (2016). Comparable reduction in Zif268 levels and cytochrome oxidase activity in the retrosplenial cortex following mammillothalamic tract lesions. Neuroscience.

[bib0315] Garden D.L., Massey P.V., Caruana D.A., Johnson B., Warburton E.C., Aggleton J.P., Bashir Z.I. (2009). Anterior thalamic lesions stop synaptic plasticity in retrosplenial cortex slices: expanding the pathology of diencephalic amnesia. Brain.

[bib0320] Geisler C., Robbe D., Zugaro M., Sirota A., Buzsaki G. (2007). Hippocampal place cell assemblies are speed-controlled oscillators. Proc. Natl. Acad. Sci. U. S. A..

[bib0325] Gentilini M., De Renzi E., Crisi G. (1987). Bilateral paramedian thalamic artery infarcts: report of eight cases. J. Neurol. Neurosurg. Psychiatry.

[bib0330] Gois Z., Tort A.B.L. (2018). Characterizing speed cells in the rat hippocampus. Cell Rep..

[bib0335] Gonzalo-Ruiz A., Sanz J.M., Lieberman A.R. (1996). Immunohistochemical studies of localization and co-localization of glutamate, aspartate and GABA in the anterior thalamic nuclei, retrosplenial granular cortex, thalamic reticular nucleus and mammillary nuclei of the rat. J. Chem. Neuroanat..

[bib0340] Gonzalo-Ruiz A., Romero J.C., Sanz J.M., Morte L. (1999). Localization of amino acids, neuropeptides and cholinergic neurotransmitter markers in identified projections from the mesencephalic tegmentum to the mammillary nuclei of the rat. J. Chem. Neuroanat..

[bib0345] Harding A., Halliday G., Caine D., Kril J. (2000). Degeneration of anterior thalamic nuclei differentiates alcoholics with amnesia. Brain.

[bib0350] Harland B., Grieves R.M., Bett D., Stentiford R., Wood E.R., Dudchenko P.A. (2017). Lesions of the head direction cell system increase hippocampal place field repetition. Curr. Biol..

[bib0355] Hasselmo M.E., Bodelon C., Wyble B.P. (2002). A proposed function for hippocampal theta rhythm: separate phases of encoding and retrieval enhance reversal of prior learning. Neural Comput..

[bib0360] Hayakawa T., Zyo K. (1989). Retrograde double-labeling study of the mammillothalamic and the mammillotegmental projections in the rat. J. Comp. Neurol..

[bib0365] Hayakawa T., Zyo K. (1991). Quantitative and ultrastructural study of ascending projections to the medial mammillary nucleus in the rat. Anat. Embryol..

[bib0370] Hildebrandt H., Muller S., Bussmann-Mork B., Goebel S., Eilers N. (2001). Are some memory deficits unique to lesions of the mammillary bodies?. J. Clin. Exp. Neuropsych..

[bib0375] Hindley E.L., Nelson A.J., Aggleton J.P., Vann S.D. (2014). Dysgranular retrosplenial cortex lesions in rats disrupt cross-modal object recognition. Learn. Mem..

[bib0380] Hindley E.L., Nelson A.J., Aggleton J.P., Vann S.D. (2014). The rat retrosplenial cortex is required when visual cues are used flexibly to determine location. Behav. Brain Res..

[bib0385] Hinman J.R., Penley S.C., Long L.L., Escabi M.A., Chrobak J.J. (2011). Septotemporal variation in dynamics of theta: speed and habituation. J. Neurophysiol..

[bib0390] Hirase H., Czurko A., Csicsvari J., Buzsaki G. (1999). Firing rate and theta-phase coding by hippocampal pyramidal neurons during ‘space clamping’. Eur. J. Neurosci..

[bib0395] Hodges J.R., McCarthy R.A. (1993). Autobiographical amnesia resulting from bilateral paramedian thalamic infarction. A case study in cognitive neurobiology. Brain.

[bib0400] Hoover W.B., Vertes R.P. (2007). Anatomical analysis of afferent projections to the medial prefrontal cortex in the rat. Brain Struct. Funct..

[bib0405] Hu H., Vervaeke K., Storm J.F. (2002). Two forms of electrical resonance at theta frequencies, generated by M-current, h-current and persistent Na+ current in rat hippocampal pyramidal cells. J. Physiol..

[bib0410] Huang L.W., Simonnet J., Nassar M., Richevaux L., Lofredi R., Fricker D. (2017). Laminar localization and projection-specific properties of presubicular neurons targeting the lateral mammillary nucleus, thalamus, or medial entorhinal cortex. eNeuro.

[bib0415] Hunkin N.M., Parkin A.J. (1993). Recency judgements in Wernicke-Korsakoff and post-encephalitic amnesia: influences of proactive interference and retention interval. Cortex.

[bib0420] Hunkin N.M., Awad M., Mayes A.R. (2015). Memory for between-list and within-list information in amnesic patients with temporal lobe and diencephalic lesions. J. Neuropsychol..

[bib0425] Huppert F.A., Piercy M. (1976). Recognition memory in amnesic patients: effect of temporal context and familiarity of material. Cortex.

[bib0430] Huppert F.A., Piercy M. (1977). Recognition memory in amnesic patients: a defect of acquisition?. Neuropsychologia.

[bib0435] Huppert F.A., Piercy M. (1978). The role of trace strength in recency and frequency judgements by amnesic and control subjects. Q. J. Exp. Psychol..

[bib0440] Hutcheon B., Yarom Y. (2000). Resonance, oscillation and the intrinsic frequency preferences of neurons. Trends Neurosci..

[bib0445] Huxter J., Burgess N., O’Keefe J. (2003). Independent rate and temporal coding in hippocampal pyramidal cells. Nature.

[bib0450] Jankowski M.M., Passecker J., Islam M.N., Vann S., Erichsen J.T., Aggleton J.P., O’Mara S.M. (2015). Evidence for spatially-responsive neurons in the rostral thalamus. Front. Behav. Neurosci..

[bib0455] Jeewajee A., Lever C., Burton S., O’Keefe J., Burgess N. (2008). Environmental novelty is signaled by reduction of the hippocampal theta frequency. Hippocampus.

[bib0460] Jenkins T.A., Vann S.D., Amin E., Aggleton J.P. (2004). Anterior thalamic lesions stop immediate early gene activation in selective laminae of the retrosplenial cortex: evidence of covert pathology in rats?. Eur. J. Neurosci..

[bib0465] Jessberger S., Clark R.E., Broadbent N.J., Clemenson G.D., Consiglio A., Lie D.C., Squire L.R., Gage F.H. (2009). Dentate gyrus-specific knockdown of adult neurogenesis impairs spatial and object recognition memory in adult rats. Learn. Mem..

[bib0470] Johnson C.T., Olton D.S., Gage F.H., Jenko P.G. (1977). Damage to hippocampus and hippocampal connections: effects on DRL and spontaneous alternation. J. Comp. Physiol. Psychol..

[bib0475] Kanter B.R., Lykken C.M., Avesar D., Weible A., Dickinson J., Dunn B., Borgesius N.Z., Roudi Y., Kentros C.G. (2017). A novel mechanism for the grid-to-place cell transformation revealed by transgenic depolarization of medial entorhinal cortex layer II. Neuron.

[bib0480] Kapur N., Cermak L.S. (1994). The mammillary bodies revisited: their role in human memory functioning. Neuropsychological Explorations of Memory and Cognition: Essays in Honor of Nelson Butters. Critical Issues in Neuropsychology.

[bib0485] Kapur N., Thompson S., Cook P., Lang D., Brice J. (1996). Anterograde but not retrograde memory loss following combined mammillary body and medial thalamic lesions. Neuropsychologia.

[bib0490] Keene C.S., Bucci D.J. (2008). Contributions of the retrosplenial and posterior parietal cortices to cue-specific and contextual fear conditioning. Behav. Neurosci..

[bib0495] Kentros C., Hargreaves E., Hawkins R.D., Kandel E.R., Shapiro M., Muller R.V. (1998). Abolition of long-term stability of new hippocampal place cell maps by NMDA receptor blockade. Science.

[bib0500] Kesner R.P., Hopkins R.O. (2001). Short-term memory for duration and distance in humans: role of the hippocampus. Neuropsychology.

[bib0505] Kessels R.P., Kopelman M.D. (2012). Context memory in Korsakoff’s syndrome. Neuropsychol. Rev..

[bib0510] Kim E., Ku J., Namkoong K., Lee W., Lee K.S., Park J., Lee S.Y., Kim J., Kim S.I., Jung Y. (2009). Mammillothalamic functional connectivity and memory function in Wernicke’s encephalopathy. Brain.

[bib0515] Kinnavane L., Vann S.D., Nelson A.J.D., O’Mara S.M., Aggleton J.P. (2018). Collateral projections innervate the mammillary bodies and retrosplenial cortex: a new category of hippocampal cells. eNeuro.

[bib0520] Kirk I.J., McNaughton N. (1991). Supramammillary cell firing and hippocampal rhythmical slow activity. Neuroreport.

[bib0525] Kirk I.J., Oddie S.D., Konopacki J., Bland B.H. (1996). Evidence for differential control of posterior hypothalamic, supramammillary, and medial mammillary theta-related cellular discharge by ascending and descending pathways. J. Neurosci..

[bib0530] Kocsis B., Vertes R.P. (1994). Characterization of neurons of the supramammillary nucleus and mammillary body that discharge rhythmically with the hippocampal theta rhythm in the rat. J. Neurosci..

[bib0535] Kocsis B., Vertes R.P. (1997). Phase relations of rhythmic neuronal firing in the supramammillary nucleus and mammillary body to the hippocampal theta activity in urethane anesthetized rats. Hippocampus.

[bib0540] Kocsis B., Di Prisco G.V., Vertes R.P. (2001). Theta synchronization in the limbic system: the role of Gudden’s tegmental nuclei. Eur. J. Neurosci..

[bib0545] Kopelman M.D., Stanhope N., Kingsley D. (1997). Temporal and spatial context memory in patients with focal frontal, temporal lobe, and diencephalic lesions. Neuropsychologia.

[bib0550] Kopelman M.D., Thomson A.D., Guerrini I., Marshall E.J. (2009). The Korsakoff syndrome: clinical aspects, psychology and treatment. Alcohol Alcohol..

[bib0555] Korsakoff S.S., Banks W.P., Karam S.J. (1996). Medico-psychological study of a memory disorder. Conscious. Cogn..

[bib0560] Kraus B.J., Robinson R.J., White J.A., Eichenbaum H., Hasselmo M.E. (2013). Hippocampal "time cells": time versus path integration. Neuron.

[bib0565] Lasztoczi B., Klausberger T. (2016). Hippocampal place cells couple to three different gamma oscillations during place field traversal. Neuron.

[bib0570] Law L.M., Smith D.M. (2012). The anterior thalamus is critical for overcoming interference in a context-dependent odor discrimination task. Behav. Neurosci..

[bib0575] Lee M.G., Chrobak J.J., Sik A., Wiley R.G., Buzsaki G. (1994). Hippocampal theta activity following selective lesion of the septal cholinergic system. Neuroscience.

[bib0580] MacDonald C.J., Lepage K.Q., Eden U.T., Eichenbaum H. (2011). Hippocampal "time cells" bridge the gap in memory for discontiguous events. Neuron.

[bib0585] Macdougall J.M., Van Hoesen G.W., Mitchell J.C. (1969). Anatomical organization of septal projections in maintenance of DRL behavior in rats. J. Comp. Physiol. Psychol..

[bib0590] Mao D., Kandler S., McNaughton B.L., Bonin V. (2017). Sparse orthogonal population representation of spatial context in the retrosplenial cortex. Nat. Commun..

[bib0595] Mao D., Neumann A.R., Sun J., Bonin V., Mohajerani M.H., McNaughton B.L. (2018). Hippocampus-dependent emergence of spatial sequence coding in retrosplenial cortex. Proc. Natl. Acad. Sci..

[bib0600] Marchand A., Faugere A., Coutureau E., Wolff M. (2014). A role for anterior thalamic nuclei in contextual fear memory. Brain Struct. Funct..

[bib0605] Marion J.F., Yang C., Caqueret A., Boucher F., Michaud J.L. (2005). Sim1 and Sim2 are required for the correct targeting of mammillary body axons. Development.

[bib0610] Mathiasen M.L., Amin E., Nelson A.J.D., Dillingham C.M., O’Mara S.M., Aggleton J.P. (2019). Separate cortical and hippocampal cell populations target the rat nucleus reuniens and mammillary bodies. Eur. J. Neurosci..

[bib0615] Mau W., Sullivan D.W., Kinsky N.R., Hasselmo M.E., Howard M.W., Eichenbaum H. (2018). The same hippocampal CA1 population simultaneously codes temporal information over multiple timescales. Curr. Biol..

[bib0620] Maurer A.P., Vanrhoads S.R., Sutherland G.R., Lipa P., McNaughton B.L. (2005). Self-motion and the origin of differential spatial scaling along the septo-temporal axis of the hippocampus. Hippocampus.

[bib0625] Mayes A.R., Downes J.J. (1997). What do theories of the functional deficit(s) underlying amnesia have to explain?. Memory.

[bib0630] Mayes A.R., Meudell P.R., Pickering A. (1985). Is organic amnesia caused by a selective deficit in remembering contextual information?. Cortex.

[bib0635] McNaughton B.L., Barnes C.A., O’Keefe J. (1983). The contributions of position, direction, and velocity to single unit activity in the hippocampus of freely-moving rats. Exp. Brain Res..

[bib0640] Mehta M.R., Barnes C.A., McNaughton B.L. (1997). Experience-dependent, asymmetric expansion of hippocampal place fields. Proc. Natl. Acad. Sci..

[bib0645] Mehta M.R., Quirk M.C., Wilson M.A. (2000). Experience-dependent asymmetric shape of hippocampal receptive fields. Neuron.

[bib0650] Meudell P.R., Mayes A.R., Ostergaard A., Pickering A. (1985). Recency and frequency judgements in alcoholic amnesics and normal people with poor memory. Cortex.

[bib0655] Miao C., Cao Q., Ito H.T., Yamahachi H., Witter M.P., Moser M.B., Moser E.I. (2015). Hippocampal remapping after partial inactivation of the medial entorhinal cortex. Neuron.

[bib0660] Milczarek M.M., Vann S.D. (2020). The retrosplenial cortex and long-term spatial memory: from the cell to the network. Curr. Opin. Behav. Sci..

[bib0665] Miller S.M., Sahay A. (2019). Functions of adult-born neurons in hippocampal memory interference and indexing. Nat. Neurosci..

[bib0670] Milczarek M.M., Vann S.D., Sengpiel F. (2018). Spatial Memory Engram in the Mouse Retrosplenial Cortex. Curr. Biol..

[bib0675] Miller A.M.P., Mau W., Smith D.M. (2019). Retrosplenial cortical representations of space and future goal locations develop with learning. Curr. Biol..

[bib0680] Mitchell A.S., Dalrymple-Alford J.C. (2005). Dissociable memory effects after medial thalamus lesions in the rat. Eur. J. Neurosci..

[bib0685] Molavi M., Vann S.D., de Vries L.S., Groenendaal F., Lequin M. (2019). Signal change in the mammillary bodies after perinatal asphyxia. AJNR Am. J. Neuroradiol..

[bib0690] Moran J.P., Dalrymple-Alford J.C. (2003). Perirhinal cortex and anterior thalamic lesions: comparative effects on learning and memory. Behav. Neurosci..

[bib0695] Muller R.U., Bostock E., Taube J.S., Kubie J.L. (1994). On the directional firing properties of hippocampal place cells. J. Neurosci..

[bib0700] Nassar M., Simonnet J., Huang L.W., Mathon B., Cohen I., Bendels M.H.K., Beraneck M., Miles R., Fricker D. (2018). Anterior thalamic excitation and feedforward inhibition of presubicular neurons projecting to medial entorhinal cortex. J. Neurosci..

[bib0705] Nelson A.J., Vann S.D. (2014). Mammilliothalamic tract lesions disrupt tests of visuo-spatial memory. Behav. Neurosci..

[bib0710] Nelson A.J.D., Vann S.D. (2017). The importance of mammillary body efferents for recency memory: towards a better understanding of diencephalic amnesia. Brain Struct. Funct..

[bib0715] O’Keefe J., Dostrovsky J. (1971). The hippocampus as a spatial map. Preliminary evidence from unit activity in the freely-moving rat. Brain Res..

[bib0720] O’Keefe J., Recce M.L. (1993). Phase relationship between hippocampal place units and the EEG theta rhythm. Hippocampus.

[bib0725] Palombo D.J., Keane M.M., Verfaellie M. (2016). Does the hippocampus keep track of time?. Hippocampus.

[bib0730] Pan W.X., McNaughton N. (1997). The medial supramammillary nucleus, spatial learning and the frequency of hippocampal theta activity. Brain Res..

[bib0735] Parker A., Gaffan D. (1997). The effect of anterior thalamic and cingulate cortex lesions on object-in-place memory in monkeys. Neuropsychologia.

[bib0740] Parker A., Gaffan D. (1997). Mamillary body lesions in monkeys impair object-in-place memory: functional unity of the fornix-mamillary system. J. Cognit. Neurosci..

[bib0745] Parkin A.J., Hunkin N.M. (1993). Impaired temporal context memory on anterograde but not retrograde tests in the absence of frontal pathology. Cortex.

[bib0750] Pastalkova E., Itskov V., Amarasingham A., Buzsáki G. (2008). Internally generated cell assembly sequences in the rat hippocampus. Science.

[bib0755] Paterlini M., Revilla V., Grant A.L., Wisden W. (2000). Expression of the neuronal calcium sensor protein family in the rat brain. Neuroscience.

[bib0760] Perry J.C., Pakkenberg B., Vann S.D. (2019). Striking reduction in neurons and glial cells in anterior thalamic nuclei of older patients with Down syndrome. Neurobiol. Aging.

[bib0765] Pitel A.L., Beaunieux H., Witkowski T., Vabret F., de la Sayette V., Viader F., Desgranges B., Eustache F. (2008). Episodic and working memory deficits in alcoholic Korsakoff patients: the continuity theory revisited. Alcohol. Clin. Exp. Res..

[bib0770] Postma A., Van Asselen M., Keuper O., Wester A.J., Kessels R.P. (2006). Spatial and temporal order memory in Korsakoff patients. J. Int. Neuropsychol. Soc..

[bib0775] Poucet B., Hok V. (2017). Remembering goal locations. Curr. Opin. Behav. Sci..

[bib0780] Powell A.L., Vann S.D., Olarte-Sanchez C.M., Kinnavane L., Davies M., Amin E., Aggleton J.P., Nelson A.J.D. (2017). The retrosplenial cortex and object recency memory in the rat. Eur. J. Neurosci..

[bib0785] Powell A., Connelly W.M., Vasalauskaite A., Nelson A.J.D., Vann S.D., Aggleton J.P., Sengpiel F., Ranson A. (2020). Stable encoding of visual cues in the mouse retrosplenial cortex. Cereb. Cortex.

[bib0790] Radyushkin K., Anokhin K., Meyer B.I., Jiang Q., Alvarez-Bolado G., Gruss P. (2005). Genetic ablation of the mammillary bodies in the Foxb1 mutant mouse leads to selective deficit of spatial working memory. Eur. J. Neurosci..

[bib0795] Ramirez J.J., Martin C., McQuilkin M.L., MacDonald K.A., Valbuena M., O’Connell J.M. (1995). Bilateral entorhinal cortex lesions impair DRL performance in rats. Psychobiology.

[bib0800] Reed L.J., Lasserson D., Marsden P., Stanhope N., Stevens T., Bello F., Kingsley D., Colchester A., Kopelman M.D. (2003). FDG-PET findings in the Wernicke-Korsakoff syndrome. Cortex.

[bib0805] Rolls E.T., Mills P. (2019). The generation of time in the hippocampal memory system. Cell Rep..

[bib0810] Rosenstock J., Field T.D., Greene E. (1977). The role of mammillary bodies in spatial memory. Exp. Neurol..

[bib0815] Rowland D.C., Yanovich Y., Kentros C.G. (2011). A stable hippocampal representation of a space requires its direct experience. Proc. Natl. Acad. Sci. U. S. A..

[bib0820] Roy D.S., Kitamura T., Okuyama T., Ogawa S.K., Sun C., Obata Y., Yoshiki A., Tonegawa S. (2017). Distinct neural circuits for the formation and retrieval of episodic memories. Cell.

[bib0825] Ruan M., Young C.K., McNaughton N. (2017). Bi-directional theta modulation between the septo-hippocampal system and the mammillary area in free-moving rats. Front. Neural Circuits.

[bib0830] Samsonovich A., McNaughton B.L. (1997). Path integration and cognitive mapping in a continuous attractor neural network model. J. Neurosci..

[bib0835] Schomburg E.W., Fernandez-Ruiz A., Mizuseki K., Berenyi A., Anastassiou C.A., Koch C., Buzsaki G. (2014). Theta phase segregation of input-specific gamma patterns in entorhinal-hippocampal networks. Neuron.

[bib0840] Seki M., Zyo K. (1984). Anterior thalamic afferents from the mamillary body and the limbic cortex in the rat. J. Comp. Neurol..

[bib0845] Senior T.J., Huxter J.R., Allen K., Neill J., Csicsvari J. (2008). Gamma oscillatory firing reveals distinct populations of pyramidal cells in the CA1 region of the hippocampus. J. Neurosci..

[bib0850] Sharp P.E., Koester K. (2008). Lesions of the mammillary body region alter hippocampal movement signals and theta frequency: implications for path integration models. Hippocampus.

[bib0855] Sharp P.E., Turner-Williams S. (2005). Movement-related correlates of single-cell activity in the medial mammillary nucleus of the rat during a pellet-chasing task. J. Neurophysiol..

[bib0860] Shen C.L. (1983). Efferent projections from the mammillary complex of the guinea pig: an autoradiographic study. Brain Res. Bull..

[bib0865] Sherman S.M., Guillery R.W. (1998). On the actions that one nerve cell can have on another: distinguishing “drivers” from “modulators”. Proc. Natl. Acad. Sci. U. S. A..

[bib0870] Shibata H. (1988). A direct projection from the entorhinal cortex to the mammillary nuclei in the rat. Neurosci. Lett..

[bib0875] Shibata H. (1989). Descending projections to the mammillary nuclei in the rat, as studied by retrograde and anterograde transport of wheat germ agglutinin-horseradish peroxidase. J. Comp. Neurol..

[bib0880] Shibata H. (1992). Topographic organization of subcortical projections to the anterior thalamic nuclei in the rat. J. Comp. Neurol..

[bib0885] Shibata H. (1993). Direct projections from the anterior thalamic nuclei to the retrohippocampal region in the rat. J. Comp. Neurol..

[bib0890] Shibata H. (1993). Efferent projections from the anterior thalamic nuclei to the cingulate cortex in the rat. J. Comp. Neurol..

[bib0895] Shibata H., Kato A. (1993). Topographic relationship between anteromedial thalamic nucleus neurons and their cortical terminal fields in the rat. Neurosci. Res..

[bib0900] Shim I., Wirtshafter D. (1996). Fos-like immunoreactivity in the mamillary body and thalamus following injections of muscimol into the ventral tegmental nucleus of Gudden in the rat. Brain Res..

[bib0905] Shoqeirat M.A., Mayes A.R. (1991). Disproportionate incidental spatial-memory and recall deficits in amnesia. Neuropsychologia.

[bib0910] Shors T.J. (2008). From stem cells to grandmother cells: how neurogenesis relates to learning and memory. Cell Stem Cell.

[bib0915] Sinden J.D., Rawlins J.N., Gray J.A., Jarrard L.E. (1986). Selective cytotoxic lesions of the hippocampal formation and DRL performance in rats. Behav. Neurosci..

[bib0920] Smith R.F., Schmaltz L.W. (1979). Acquisition of appetitively and aversively motivated tasks in rats following lesions of the mammillary bodies. Physiol. Psychol..

[bib0925] Stark E., Eichler R., Roux L., Fujisawa S., Rotstein H.G., Buzsaki G. (2013). Inhibition-induced theta resonance in cortical circuits. Neuron.

[bib0930] Stone S.S., Teixeira C.M., Devito L.M., Zaslavsky K., Josselyn S.A., Lozano A.M., Frankland P.W. (2011). Stimulation of entorhinal cortex promotes adult neurogenesis and facilitates spatial memory. J. Neurosci..

[bib0935] Stuss D.T., Guberman A., Nelson R., Larochelle S. (1988). The neuropsychology of paramedian thalamic infarction. Brain Cogn..

[bib0940] Sullivan E.V., Pfefferbaum A. (2009). Neuroimaging of the Wernicke-Korsakoff syndrome. Alcohol Alcohol..

[bib0945] Sun Y., Jin S., Lin X., Chen L., Qiao X., Jiang L., Zhou P., Johnston K.G., Golshani P., Nie Q., Holmes T.C., Nitz D.A., Xu X. (2019). CA1-projecting subiculum neurons facilitate object-place learning. Nat. Neurosci..

[bib0950] Sweeney-Reed C.M., Zaehle T., Voges J., Schmitt F.C., Buentjen L., Kopitzki K., Hinrichs H., Heinze H.J., Rugg M.D., Knight R.T., Richardson-Klavehn A. (2015). Thalamic theta phase alignment predicts human memory formation and anterior thalamic cross-frequency coupling. eLife.

[bib0955] Sweeney-Reed C.M., Zaehle T., Voges J., Schmitt F.C., Buentjen L., Kopitzki K., Richardson-Klavehn A., Hinrichs H., Heinze H.J., Knight R.T., Rugg M.D. (2016). Pre-stimulus thalamic theta power predicts human memory formation. NeuroImage.

[bib0960] Sweeney-Reed C.M., Zaehle T., Voges J., Schmitt F.C., Buentjen L., Borchardt V., Walter M., Hinrichs H., Heinze H.J., Rugg M.D., Knight R.T. (2017). Anterior thalamic high frequency band activity is coupled with theta oscillations at rest. Front. Hum. Neurosci..

[bib0965] Szabo N.E., Haddad-Tovolli R., Zhou X., Alvarez-Bolado G. (2015). Cadherins mediate sequential roles through a hierarchy of mechanisms in the developing mammillary body. Front. Neuroanat..

[bib0970] Talk A., Kang E., Gabriel M. (2004). Independent generation of theta rhythm in the hippocampus and posterior cingulate cortex. Brain Res..

[bib0975] Talley E.M., Cribbs L.L., Lee J.H., Daud A., Perez-Reyes E., Bayliss D.A. (1999). Differential distribution of three members of a gene family encoding low voltage-activated (T-type) calcium channels. J. Neurosci..

[bib0980] Tanaka Y., Miyazawa Y., Akaoka F., Yamada T. (1997). Amnesia following damage to the mammillary bodies. Neurology.

[bib0985] Tielemans N.S., Hendriks M.P., Talamini L., Wester A.J., Meeter M., Kessels R.P. (2012). Facilitation of memory by contextual cues in patients with diencephalic or medial temporal lobe dysfunction. Neuropsychologia.

[bib0990] Toda H., Hamani C., Fawcett A.P., Hutchison W.D., Lozano A.M. (2008). The regulation of adult rodent hippocampal neurogenesis by deep brain stimulation. J. Neurosurg..

[bib0995] Tonkiss J., Rawlins J.N. (1992). Mammillary body lesions and restricted subicular output lesions produce long-lasting DRL performance impairments in rats. Exp. Brain Res..

[bib1000] Tronel S., Charrier V., Sage C., Maitre M., Leste-Lasserre T., Abrous D.N. (2015). Adult-born dentate neurons are recruited in both spatial memory encoding and retrieval. Hippocampus.

[bib1005] Tsanov M., Chah E., Vann S.D., Reilly R.B., Erichsen J.T., Aggleton J.P., O’Mara S.M. (2011). Theta-modulated head direction cells in the rat anterior thalamus. J. Neurosci..

[bib1010] Tsanov M., Chah E., Wright N., Vann S.D., Reilly R., Erichsen J.T., Aggleton J.P., O’Mara S.M. (2011). Oscillatory entrainment of thalamic neurons by theta rhythm in freely moving rats. J. Neurophysiol..

[bib1015] Tsao A., Sugar J., Lu L., Wang C., Knierim J.J., Moser M.B., Moser E.I. (2018). Integrating time from experience in the lateral entorhinal cortex. Nature.

[bib1020] Tsuchiya R., Takahashi K., Liu F.C., Takahashi H. (2009). Aberrant axonal projections from mammillary bodies in Pax6 mutant mice: possible roles of Netrin-1 and Slit 2 in mammillary projections. J. Neurosci. Res..

[bib1025] Valverde F., Garcia C., Lopez-Mascaraque L., De Carlos J.A. (2000). Development of the mammillothalamic tract in normal and Pax-6 mutant mice. J. Comp. Neurol..

[bib1030] van der Kooy D., Kuypers H.G., Catsman-Berrevoets C.E. (1978). Single mammillary body cells with divergent axon collaterals. Demonstration by a simple, fluorescent retrograde double labeling technique in the rat. Brain Res..

[bib1035] Van der Werf Y.D., Jolles J., Witter M.P., Uylings H.B. (2003). Contributions of thalamic nuclei to declarative memory functioning. Cortex.

[bib1040] van Groen T., Wyss J.M. (1990). The postsubicular cortex in the rat: characterization of the fourth region of the subicular cortex and its connections. Brain Res..

[bib1045] van Groen T., Kadish I., Wyss J.M. (1999). Efferent connections of the anteromedial nucleus of the thalamus of the rat. Brain Res. Brain Res. Rev..

[bib1050] Van Groen T., Wyss J.M. (1995). Projections from the anterodorsal and anteroventral nucleus of the thalamus to the limbic cortex in the rat. J. Comp. Neurol..

[bib1055] Van Groen T., Wyss J.M. (2003). Connections of the retrosplenial granular b cortex in the rat. J. Comp. Neurol..

[bib1060] van Rijckevorsel K., Abu Serieh B., de Tourtchaninoff M., Raftopoulos C. (2005). Deep EEG recordings of the mammillary body in epilepsy patients. Epilepsia.

[bib1065] Vanderwolf C.H. (1969). Hippocampal electrical activity and voluntary movement in the rat. Electroencephalogr. Clin. Neurophysiol..

[bib1070] Vann S.D. (2005). Transient spatial deficit associated with bilateral lesions of the lateral mammillary nuclei. Eur. J. Neurosci..

[bib1075] Vann S.D. (2011). A role for the head-direction system in geometric learning. Behav. Brain Res..

[bib1080] Vann S.D. (2013). Dismantling the Papez circuit for memory in rats. eLife.

[bib1085] Vann S.D. (2018). Lesions within the head direction system reduce retrosplenial c-fos expression but do not impair performance on a radial-arm maze task. Behav. Brain Res..

[bib1090] Vann S.D., Aggleton J.P. (2003). Evidence of a spatial encoding deficit in rats with lesions of the mammillary bodies or mammillothalamic tract. J. Neurosci..

[bib1095] Vann S.D., Aggleton J.P. (2005). Selective dysgranular retrosplenial cortex lesions in rats disrupt allocentric performance of the radial-arm maze task. Behav. Neurosci..

[bib1100] Vann S.D., Albasser M.M. (2009). Hippocampal, retrosplenial, and prefrontal hypoactivity in a model of diencephalic amnesia: evidence towards an interdependent subcortical-cortical memory network. Hippocampus.

[bib1105] Vann S.D., Honey R.C., Aggleton J.P. (2003). Lesions of the mammillothalamic tract impair the acquisition of spatial but not nonspatial contextual conditional discriminations. Eur. J. Neurosci..

[bib1110] Vann S.D., Aggleton J.P., Maguire E.A. (2009). What does the retrosplenial cortex do?. Nat. Rev. Neurosci..

[bib1115] Vann S.D., Tsivilis D., Denby C.E., Quamme J.R., Yonelinas A.P., Aggleton J.P., Montaldi D., Mayes A.R. (2009). Impaired recollection but spared familiarity in patients with extended hippocampal system damage revealed by 3 convergent methods. Proc. Acad. Nat. Sci..

[bib1120] Vann S.D., Erichsen J.T., O’Mara S.M., Aggleton J.P. (2011). Selective disconnection of the hippocampal formation projections to the mammillary bodies produces only mild deficits on spatial memory tasks: implications for fornix function. Hippocampus.

[bib1125] Vertes R.P., Albo Z., Viana Di Prisco G. (2001). Theta-rhythmically firing neurons in the anterior thalamus: implications for mnemonic functions of Papez’s circuit. Neuroscience.

[bib1130] Vetreno R.P., Klintsova A., Savage L.M. (2011). Stage-dependent alterations of progenitor cell proliferation and neurogenesis in an animal model of Wernicke-Korsakoff syndrome. Brain Res..

[bib1135] Vukovic J., Borlikova G.G., Ruitenberg M.J., Robinson G.J., Sullivan R.K., Walker T.L., Bartlett P.F. (2013). Immature doublecortin-positive hippocampal neurons are important for learning but not for remembering. J. Neurosci..

[bib1140] Warburton E.C., Aggleton J.P. (1999). Differential deficits in the Morris water maze following cytotoxic lesions of the anterior thalamus and fornix transection. Behav. Brain Res..

[bib1145] Warburton E.C., Brown M.W. (2015). Neural circuitry for rat recognition memory. Behav. Brain Res..

[bib1150] Welday A.C., Shlifer I.G., Bloom M.L., Zhang K., Blair H.T. (2011). Cosine directional tuning of theta cell burst frequencies: evidence for spatial coding by oscillatory interference. J. Neurosci..

[bib1155] Wiener S.I., Paul C.A., Eichenbaum H. (1989). Spatial and behavioral correlates of hippocampal neuronal activity. J. Neurosci..

[bib1160] Winocur G., Becker S., Luu P., Rosenzweig S., Wojtowicz J.M. (2012). Adult hippocampal neurogenesis and memory interference. Behav. Brain Res..

[bib1165] Winter S.S., Wagner S.J., McMillin J.L., Wallace D.G. (2011). Mammillothalamic tract lesions disrupt dead reckoning in the rat. Eur. J. Neurosci..

[bib1170] Winter S.S., Clark B.J., Taube J.S. (2015). Spatial navigation. Disruption of the head direction cell network impairs the parahippocampal grid cell signal. Science.

[bib1175] Wirtshafter D., Stratford T.R. (1993). Evidence for GABAergic projections from the tegmental nuclei of Gudden to the mammillary body in the rat. Brain Res..

[bib1180] Wolff M., Vann S.D. (2019). The cognitive thalamus as a gateway to mental representations. J. Neurosci..

[bib1185] Wolff M., Gibb S.J., Dalrymple-Alford J.C. (2006). Beyond spatial memory: the anterior thalamus and memory for the temporal order of a sequence of odor cues. J. Neurosci..

[bib1190] Wright N.F., Erichsen J.T., Vann S.D., O’Mara S.M., Aggleton J.P. (2010). Parallel but separate inputs from limbic cortices to the mammillary bodies and anterior thalamic nuclei in the rat. J. Comp. Neurol..

[bib1195] Xu X., Sun Y., Holmes T.C., Lopez A.J. (2016). Noncanonical connections between the subiculum and hippocampal CA1. J. Comp. Neurol..

[bib1200] Yamadori T. (1973). An experimental anatomical study of the fasciculus mamillothalamicus in rats. J. Hirnforsch..

[bib1205] Zakowski W., Rowniak M., Robak A. (2014). Colocalization pattern of calbindin and cocaine- and amphetamine-regulated transcript in the mammillary body-anterior thalamic nuclei axis of the guinea pig. Neuroscience.

[bib1210] Zakowski W., Zawistowski P., Braszka L., Jurkowlaniec E. (2017). The effect of pharmacological inactivation of the mammillary body and anterior thalamic nuclei on hippocampal theta rhythm in urethane-anesthetized rats. Neuroscience.

[bib1215] Zappala A., Li Volti G., Serapide M.F., Pellitteri R., Falchi M., La Delia F., Cicirata V., Cicirata F. (2007). Expression of pannexin2 protein in healthy and ischemized brain of adult rats. Neuroscience.

[bib1220] Zeldenrust F., Wadman W.J., Englitz B. (2018). Neural coding with bursts—Current state and future perspectives. Front. Comput. Neurosci..

[bib1225] Zhao N., Zhong C., Wang Y., Zhao Y., Gong N., Zhou G., Xu T., Hong Z. (2008). Impaired hippocampal neurogenesis is involved in cognitive dysfunction induced by thiamine deficiency at early pre-pathological lesion stage. Neurobiol. Dis..

[bib1230] Zheng C., Bieri K.W., Hsiao Y.T., Colgin L.L. (2016). Spatial sequence coding differs during slow and fast gamma rhythms in the Hippocampus. Neuron.

